# Combined Immunotherapy and Hybrid Dose-Fraction Radiotherapy for Driver-Negative Metastatic NSCLC: A Phase II Trial with Parallel Preclinical Studies

**DOI:** 10.34133/cancomm.0065

**Published:** 2026-11-10

**Authors:** Ziye Li, Xiaoyang Li, Xiaoying Wei, Lejie Cao, Junqiang Zhang, Jianguo Wang, Yuan He, Anqi Hu, Lei Liu, Bing Yan, Congyu Wang, Hongxia Li, Ping Zeng, Jingjing Cheng, Anlin Wang, Jun Ma, Huailing Xia, Haibo Wu, Daqing Xia, Fengshou Jiang, Dan Xie, Dong Qian

**Affiliations:** ^1^ Department of Radiation Oncology, The First Affiliated Hospital of University of Science and Technology of China, Division of Life Sciences and Medicine, University of Science and Technology of China, Hefei, Anhui, P. R. China.; ^2^ Core Facility Center, The First Affiliated Hospital of University of Science and Technology of China, Division of Life Sciences and Medicine, University of Science and Technology of China, Hefei, Anhui, P. R. China.; ^3^ Anhui Provincial Cancer Hospital, Hefei, Anhui, P. R. China.; ^4^ Department of Respiratory and Critical Care Medicine, The First Affiliated Hospital of University of Science and Technology of China, Division of Life Sciences and Medicine, University of Science and Technology of China, Hefei, Anhui, P. R. China.; ^5^ The Center of Radiation Oncology, Guoyang County People’s Hospital, Bozhou, Anhui, P. R. China.; ^6^ Department of Oncology, The Third Affiliated Hospital of Anhui Medical University, Hefei, Anhui, P. R. China.; ^7^ Department of Pathology, The First Affiliated Hospital of University of Science and Technology of China, Division of Life Sciences and Medicine, University of Science and Technology of China, Hefei, Anhui, P. R. China.; ^8^ Department of Medical Oncology, The First Affiliated Hospital of University of Science and Technology of China, Division of Life Sciences and Medicine, University of Science and Technology of China, Hefei, Anhui, P. R. China.; ^9^ State Key Laboratory of Oncology in South China, Guangdong Provincial Clinical Research Center for Cancer, Sun Yat-sen University Cancer Center, Guangzhou, Guangdong, P. R. China.; ^10^ Anhui Provincial Engineering Research Center for Radiation Oncology (AERRO), Hefei, Anhui, P. R. China.

## Abstract

**Background:** Immune checkpoint inhibitor (ICI)-based chemoimmunotherapy has become a standard first-line treatment for patients with driver-negative metastatic non-small cell lung cancer (NSCLC), but the clinical benefit is limited. Radiotherapy has potential antitumor immunomodulatory effects, but the optimal dose and fractionation remain unclear. **Methods:** This single-arm, multicenter, prospective, phase II trial enrolled patients with stage IV driver-negative NSCLC who received first-line chemoimmunotherapy. During the ICI maintenance phase, patients received hybrid dose-fraction radiotherapy (Hybrid-RT): stereotactic ablative body radiotherapy (SABR) to targetable lesions and low-dose radiotherapy (LDRT) to other sites. Patients restaged to stage III disease after chemoimmunotherapy received short-course hypofractionated radiotherapy to all residual lesions. The primary endpoint was 1-year progression-free survival (PFS) rate. The secondary endpoints included 1-year overall survival (OS) rate, objective response rate, and safety. Parallel preclinical mechanistic studies were performed in a murine model. **Results:** A total of 83 patients were enrolled from 2022 May 9 to 2023 December 31. The 1-year PFS rate was 54.2% (median PFS 16.1 months; 95% confidence interval [CI] 11.4 to 19.2 months), meeting the primary endpoint. The 1-year OS rate was 83.1% (median OS 29.3 months; 95% CI 21.9 months to not reached), and treatment was well tolerated. Peripheral immune profiling showed posttreatment expansion of cytotoxic CD8^+^ T cells and natural killer (NK) cells associated with promising clinical efficacy. In the murine model, ICI plus Hybrid-RT (IHBRT) induced a division of labor between CD8^+^ T and NK cells. At LDRT lesions, it facilitated both C-X-C motif chemokine receptor 3 (Cxcr3)-mediated recruitment of cytotoxic CD8^+^ T cells and natural killer group 2 member D-mediated NK cell cytotoxicity. At SABR lesions, Cxcr3-mediated CD8^+^ T cells played a predominant role, whereas NK cells contributed minimally. Moreover, IHBRT suppressed secreted phosphoprotein 1 positive (Spp1^+^) macrophages and relieved immunosuppression. **Conclusions:** Clinically, IHBRT demonstrates promising efficacy and a manageable safety profile in driver-negative metastatic NSCLC. Preclinically, it induces a functional division of labor between CD8^+^ T and NK cells to activate antitumor immune cytotoxicity, and suppresses Spp1^+^ macrophages, thereby relieving immunosuppression. **Trial registration:** This trial was registered at ClinicalTrials.gov (Identifier: NCT05348668). Date of registration: 2022 April 26.

## Background

Non-small cell lung cancer (NSCLC) comprises heterogeneous pathological subtypes and molecular profiles [1]. From a molecular and therapeutic perspective, NSCLC can be broadly categorized into 2 groups: driver gene-positive and driver gene-negative. While immune checkpoint inhibitors (ICIs) have provided substantial clinical benefit to patients with driver gene-negative tumors, a substantial proportion of those with advanced disease still do not achieve long-term survival [2–4]. Therefore, overcoming immune resistance remains a major therapeutic challenge.

Radiotherapy plays a role in the treatment of NSCLC at almost all stages. Accumulating clinical evidence has demonstrated synergism between radiotherapy and immunotherapy [5,6], whereby radiotherapy enhances the efficacy of ICIs in both early- and advanced-stage NSCLC [7–11]. Preclinical studies indicate that radiotherapy promotes antitumor immunity by inducing immunogenic cell death (ICD) and remodeling the tumor immune microenvironment (TIME). The core mechanism by which radiotherapy exerts antitumor effects involves ionizing radiation-induced DNA damage, which activates ICD pathways. This can convert irradiated tumor cells into an in situ tumor vaccine, triggering systemic antitumor responses in nonirradiated distant lesions, a phenomenon known as the abscopal effect [12]. At the molecular level, radiotherapy-induced cytoplasmic DNA fragments activate the cyclic GMP-AMP synthase (cGAS)–stimulator of interferon genes (STING) pathway, initiating interferon (IFN)-mediated antitumor immunity [13,14]. Furthermore, radiotherapy can disrupt the immunosuppressive barrier and convert “cold” tumors into immunologically “hot” tumors, which is critical for effective cancer therapy [15].

However, the impact of radiotherapy on antitumor immunity is double-edged. Conventionally fractionated radiotherapy, widely used in clinical practice, exerts prolonged cytotoxic and suppressive effects on circulating lymphocytes [16]. Furthermore, the cGAS–STING pathway may activate the atypical nuclear factor kappa-light-chain-enhancer of activated B cells pathway in dendritic cells (DCs), promoting the infiltration of myeloid-derived suppressor cells into the TIME and reinforcing immunosuppression [17,18]. Together, these factors limit the clinical benefit of radioimmunotherapy combinations.

In stage IV NSCLC treated with radiotherapy plus immunotherapy, immunotherapy serves as the primary systemic treatment, while radiotherapy acts as a local immunomodulatory adjuvant. Hypofractionated radiotherapy, especially stereotactic ablative body radiotherapy (SABR), effectively induces ICD and enhances antitumor immune activation. Meanwhile, low-dose radiotherapy (LDRT) can remodel the TIME and increase immune cell infiltration [19–21]. Both SABR and LDRT are short regimens that reduce radiation-induced lymphopenia. Emerging evidence indicates that combining SABR and LDRT can robustly activate antitumor immunity and improve the efficacy of ICIs [22–24]. However, the optimal combination schedule of SABR, LDRT, and immunotherapy remains unclear, restricting their broad clinical use. Moreover, the detailed molecular mechanisms remain incompletely defined.

To address these gaps, we designed a phase II clinical trial to evaluate the efficacy and safety of first-line ICI combined with hybrid dose-fraction radiotherapy (Hybrid-RT), integrating SABR for eligible lesions and LDRT for SABR-unsuitable lesions, in patients with stage IV driver gene-negative NSCLC. Additionally, using single-cell RNA sequencing (scRNA-seq) and functional assays in NSCLC murine models, we preliminarily explored the mechanisms through which ICI plus Hybrid-RT (IHBRT) activates antitumor immunity.

## Materials and Methods

### Study design and participants

This single-arm, multicenter, prospective phase II clinical study was conducted at 4 medical centers across China. The clinical trial was registered at ClinicalTrials.gov under the identifier NCT05348668. Eligible patients (18 to 75 years) had histologically confirmed stage IV driver-negative NSCLC (8th edition of the Tumor–Node–Metastasis [TNM] Staging System [25]), Eastern Cooperative Oncology Group (ECOG) performance status 0 to 1, and adequate organ/bone marrow function. Exclusion criteria included driver mutations (e.g., epidermal growth factor receptor [*EGFR*], anaplastic lymphoma kinase [*ALK*], and ROS proto-oncogene 1 [*ROS1*]), pulmonary fibrosis, autoimmune disease, active infection, or serious comorbidities. All patients received 4 to 6 cycles of platinum-based doublet chemotherapy plus ICI. Patients without disease progression after comprehensive evaluation subsequently underwent Hybrid-RT during ICI maintenance. The comprehensive clinical trial protocol is provided in Data S1.

### Procedures

#### Diagnosis and staging

Eligibility to participate in the study required histologically confirmed diagnosis and completion of molecular profiling (including at least *EGFR*, *ALK*, *ROS1*, and programmed cell death ligand 1 [PD-L1] status). All patients were pre-evaluated and staged via bronchoscopy, computed tomography (CT) with contrast, contrast-enhanced magnetic resonance imaging (MRI) of the brain, and single-photon emission CT bone scan; positron emission tomography (PET)/CT or PET/MRI was optional. Histopathological diagnosis of suspicious lymph nodes was established via either thoracoscopic biopsy or endosonography-guided needle aspiration as clinically warranted.

#### First-line chemoimmunotherapy

All enrolled patients received 4 to 6 cycles of standard platinum-based doublet chemotherapy combined with ICI therapy. Specific chemotherapy regimens and ICI agents were selected at the investigators’ discretion in accordance with both the National Comprehensive Cancer Network guidelines [26] and the Chinese Society of Clinical Oncology guidelines. ICI maintenance continued for up to 24 months or until disease progression, unacceptable toxicity, or study withdrawal occurred.

#### Radiotherapy

Patients without progression after chemoimmunotherapy received Hybrid-RT during ICI maintenance, completed within 3 months. SABR-eligible lesions were treated with hypofractionated radiotherapy (24 Gy/3 fractions or 50 Gy/4 to 5 fractions), while non-SABR lesions received LDRT (6 to 15 Gy at 1.5 Gy/fraction). For patients who were restaged as stage III disease after chemoimmunotherapy, the primary tumor and regional metastatic lymph nodes received short-course hypofractionated radiotherapy with 48 Gy in 12 fractions, which achieves a biologically effective dose (BED) equivalent to conventional fractionation of 54 Gy in 27 fractions (assuming an α/β ratio of 10 Gy for NSCLC). Target selection and dose schedules are detailed in Tables S1 and S2. The simulation utilized customized vacuum immobilization with intravenous contrast-enhanced 4-dimensional CT. The internal target volume (ITV) was contoured on maximum intensity projection reconstructions, with the planning target volume defined as the ITV plus a 5-mm isotropic margin. All treatments followed predefined plans, minimizing inter-/intra-fractional errors.

#### Monitoring and follow-up

The primary endpoint was the 1-year progression-free survival (PFS) rate. PFS was defined as the time from initiation of first-line chemoimmunotherapy to disease progression per Response Evaluation Criteria in Solid Tumors (RECIST v1.1) or death from any cause, whichever occurred first. Patients without documented disease progression or death at the time of data cutoff were censored at the date of their last disease assessment. The secondary endpoints were median PFS, 1.5-year and 2-year PFS rates, median overall survival (OS), 1-year and 2-year OS rates, objective response rate (ORR), disease control rate (DCR), and adverse events (AEs). OS was defined as the time from initiation of first-line chemoimmunotherapy to death from any cause (living patients were censored at last follow-up). Per RECIST v1.1, ORR and DCR represented patients achieving complete or partial response, and complete/partial response or stable disease, respectively. Hybrid-RT was initiated no less than 2 weeks after the completion of initial chemoimmunotherapy. Exploratory outcomes included changes in peripheral blood lymphocyte subsets pre-radiotherapy and 3 weeks post-radiotherapy. AEs and laboratory/radiological assessments were performed weekly during chemoimmunotherapy and radiotherapy, and every 3 weeks during ICI maintenance. AEs were graded according to the Common Terminology Criteria for Adverse Events (CTCAE version 4.0). Tumor assessments per RECIST v1.1 were conducted every 9 weeks in the first year, every 3 months in the second year, and every 6 months thereafter.

### Analysis of the immune cells of the participants

Peripheral blood mononuclear cells (PBMCs) obtained from 40 patients who received Hybrid-RT (SABR + LDRT) at the First Affiliated Hospital of University of Science and Technology of China (USTC) were isolated within 2 h of EDTA anticoagulated blood collection using Ficoll-Paque (Cat. #17144002, Cytiva) density gradient centrifugation. All patients completed chemoimmunotherapy ≥2 weeks prior to Hybrid-RT. Peripheral blood was collected immediately before radiotherapy and 3 weeks after treatment completion. Immune cells were analyzed via clinical-grade flow cytometry and were defined as follows: CD3^+^ T cells (CD45^+^CD3^+^), T helper cells (Th; CD45^+^CD3^+^CD4^+^CD8^−^), granzyme B (GZMB)^+^ Th cells (CD45^+^CD3^+^CD4^+^CD8^−^GZMB^+^), CD8^+^ T cells (CD45^+^CD3^+^CD8^+^CD4^−^), cytotoxic CD8^+^ T cells (cyto-CD8^+^ T; CD45^+^CD3^+^CD8^+^CD4^−^GZMB^+^), natural killer cells (NK; CD45^+^CD3^−^CD56^dim^), and cytotoxic NK cells (cyto-NK; CD45^+^CD3^−^CD56^dim^GZMB^+^).

### Animal study

C57BL/6 mice (5 weeks old, male) were obtained from Shanghai SLAC Laboratory Animal Co., Ltd. and housed in a specific pathogen-free barrier facility at the Core Facility Center of the First Affiliated Hospital of USTC. Murine Lewis lung carcinoma (LLC) cells (Cat. #QuiCell-L214, QuiCell Biotechnology Co., Ltd.) were cultured in Dulbecco’s modified Eagle medium (DMEM) supplemented with 10% fetal bovine serum (FBS) at 37 °C with 5% CO₂. DMEM (Cat. #10566016, Gibco) and FBS (Cat. #A5669701, Gibco) were purchased from Ketu Biotech Co., Ltd. Mycoplasma contamination was routinely monitored every 2 weeks. Subcutaneous tumors (primary lesions) were established by injecting 2.5 × 10^5^ LLC cells into the right flank on day –21, and lung metastases were induced on the same day by intravenous injection of 5 × 10^5^ LLC cells via the tail vein (experimental metastasis model). From day –21 to day 0, tumors were allowed to grow without intervention.

SABR was delivered on days 1, 3, and 5; LDRT was delivered on days 7, 12, and 17. Anti-programmed death receptor 1 (anti-PD-1; Cat. #BE0146, BioXCell), anti-C-X-C motif chemokine receptor 3 (anti-Cxcr3; Cat. #BE0249, BioXCell), and anti-natural killer group 2 member D (anti-NKG2D; Cat. #BE0111, BioXCell) were administered intraperitoneally at 200 μg per mouse on days 7, 12, and 17. For irradiation, tumor-bearing mice were anesthetized with isoflurane (induction 2%, maintenance 1.5%; Cat. #HY-A0134, MedChemExpress), and the irradiation sites were fully exposed. Targeted pulmonary or subcutaneous tumors were irradiated using a rodent-specific x-ray irradiator (Cat. #SHARP 100, Raycision Medical Technology Co., Ltd.). For lung metastatic lesions, a dose of 1 Gy (1 Gy/min, 225 kV) was delivered to the entire lung, while subcutaneous tumors (primary lesions) received 8 Gy (1 Gy/min, 225 kV). Serial micro-CT imaging was performed using a micro-CT scanner (Cat. #IMAGING 100, Raycision Medical Technology Co., Ltd.) on days 0, 7, and 21. Tumor volume (*V*) was calculated as *V* = (length × width^2^)/2, where length was the longest diameter and width was the perpendicular diameter. Mice were euthanized on day 21, and tumors, lung tissues, and serum were collected for analysis.

### Histological and immunohistochemical staining

Tissue samples were fixed in 4% paraformaldehyde (Cat. #BL539A, Biosharp) for 24 h, paraffin-embedded, and sectioned at 4 μm thickness. For hematoxylin and eosin (H&E) staining, sections were deparaffinized, rehydrated, and stained according to standard protocols. For Masson’s staining, sections were stained with a Masson’s trichrome stain kit (Cat. #C0189S, Beyotime) according to the manufacturer’s instructions. For immunohistochemistry (IHC), antigen retrieval was performed using citrate buffer (pH 6.0; Cat. #BL604A, Biosharp) at 95 °C for 15 min, followed by blocking of endogenous peroxidase activity with 3% hydrogen peroxide (Cat. #abs9333, Absin). Sections were incubated overnight at 4 °C with primary antibodies against NK1.1 (Cat. #ab289542, Abcam), transforming growth factor-beta (TGF-β; Cat. #21898-1-AP, Proteintech), followed by incubation with secondary antibodies at 37 °C for 1 h, and visualized using a 3,3′-diaminobenzidine substrate kit (Cat. #ab64238, Abcam). The antibodies used for IHC staining are listed in Table S3. After hematoxylin counterstaining, staining intensity and distribution were quantified using Fiji software (ImageJ v1.53t, https://fiji.sc/) based on standardized thresholds.

### Multiplex immunofluorescence

Tissues were prepared as described for IHC. Sections were incubated overnight at 4 °C with primary antibodies against CD8 (Cat. #ab217344, Abcam), CD3 (Cat. #ab135372, Abcam), NK1.1 (Cat. #ab289542, Abcam), CXCR3 (Cat. #ab288437, Abcam), C-X-C motif chemokine ligand 10 (CXCL10, Cat. #10937-1-AP, Proteintech), GZMB (Cat. #13588-1-AP, Proteintech), F4/80 (Cat. #sc-52664, Santa Cruz), and secreted phosphoprotein 1 (SPP1, Cat. #sc-21742, Santa Cruz). After washing, sections were incubated at room temperature for 1 h with Cy3 Goat-Anti-Rabbit IgG (H + L) (Cat. #ab6939, Abcam), Alexa Fluor 488 Goat-Anti-Mouse IgG (H + L) (Cat. #ab150113, Abcam), and Cy5 Goat-Anti-Rabbit IgG (H + L) (Cat. #ab6564, Abcam). The antibodies used for multiplex immunofluorescence (mIF) staining are listed in Table S3. Sections were mounted with 4′,6-diamidino-2-phenylindole (DAPI) containing anti-fade mounting medium (Cat. #F6057, Sigma) and examined by fluorescence microscopy.

### Flow cytometry

Single-cell suspensions were prepared from lung/tumor tissues by mechanical dissociation and filtration through a 70-μm filter. The cells were washed in cold phosphate-buffered saline (PBS, Cat. #BL302A, Biosharp), resuspended at 1 × 10^7^ cells/ml, and Fc-blocked (Cat. #15660, BioLegend) prior to surface staining. To explore intracellular targets, cells were fixed/permeabilized with BD Cytofix/Cytoperm (Cat. #554714, BD Biosciences) and then stained with antibodies against cytokines/activation markers. The samples were analyzed on a BD FACSCelesta using FlowJo (v10.9, https://www.flowjo.com/docs/flowjo10). Details regarding the antibodies used are provided in Table S3.

### scRNA-seq

All tissue samples were collected on day 21. Fresh tissue samples (*n* = 3/group) were dissociated into single-cell suspensions using the GentleMACS Dissociator (Cat. #130-093-235, Miltenyi Biotec) and filtered through 30-μm strainers. Cell viability (>85%) was confirmed via Trypan blue (Cat. #T8154, Sigma-Aldrich) exclusion. The fixed cells were hybridized with species-specific probes using the Chromium X system (10x Genomics, Pleasanton). The obtained libraries were sequenced on the NovaSeq 6000 platform (Illumina; 150 bp paired-end, ≥20,000 reads/cell). The data were processed with Cell Ranger (v7.1.0, https://support.10xgenomics.com/) and analyzed using Seurat (v4.3, https://satijalab.org/seurat/), excluding low-quality cells (<500 genes/cell, >15% mitochondrial reads). Gene set enrichment analysis (GSEA) was performed using clusterProfiler (v4.6.0) in R (v4.3.1, https://www.r-project.org/) against MSigDB (https://www.gsea-msigdb.org/gsea/msigdb) hallmark gene sets; pathways with false discovery rate (FDR) < 0.25 were considered significant. The scRNA-seq analysis was performed by OE Biotech (Shanghai, China).

### CellChat analysis

Cell–cell communication networks were inferred using CellChat (v2.1.2) in R (v4.3.1, https://www.r-project.org/). The normalized expression matrix was imported and used to generate a CellChat object, followed by preprocessing to identify overexpressed genes and ligand–receptor interactions using default parameters. Communication probabilities were calculated (computeCommunProb) and filtered to exclude interactions with fewer than 10 cells (filterCommunication), and pathway-level networks were aggregated (aggregateNet). Signaling pathways were prioritized on the basis of interaction strength and network centrality.

### Cytokine determination via Luminex

Serum levels of cytokines were quantified using the Luminex X-200 system (Luminex) according to the manufacturer’s instructions (LX-MultiDTH-10, LabEx Co.).

### Statistical analysis

Clinical efficacy analyses followed the intention-to-treat (ITT) principle. Based on historical control data [27–29], a sample size of 74 patients (including an estimated 10% dropout rate) was calculated to provide 80% power to detect an improvement in the 1-year PFS rate from 35% to 50%, using a one-sided *α* level of 0.05. The cutoff date was 2025 September 30. For the clinical trial, survival outcomes were measured from first-line chemoimmunotherapy initiation. The comprehensive clinical statistical analysis plan is provided in the Data S2. SPP1^+^ macrophage-associated signature scores were calculated using bulk RNA-sequencing data from The Cancer Genome Atlas (TCGA) lung adenocarcinoma (LUAD) and lung squamous cell carcinoma (LUSC) cohorts (https://www.cancer.gov/tcga). Single-sample gene set enrichment analysis (ssGSEA) was performed using a marker gene set derived from the GSE127465 single-cell RNA-sequencing dataset. These scores reflect transcriptional enrichment. The optimal cutoff for the SPP1^+^ macrophage-associated signature score was determined using the survminer R package (v0.5.1). Patients were classified into high-score (≥ cutoff) and low-score (< cutoff) groups. All experiments were independently replicated 3 times with consistent results, and representative data are shown. The statistical tests used for data analysis included Student’s *t* test, one-way repeated-measures analysis of variance (ANOVA), the *χ*^2^ test, and the log-rank test; differential gene expression was assessed using the Wilcoxon rank-sum test with Benjamini–Hochberg correction. Survival curves were estimated using the Kaplan–Meier method and compared using the log-rank test. The data were analyzed using SPSS (v25.0, https://www.ibm.com/docs/spss-statistics) and R (v4.3.1, https://www.r-project.org/). Statistical significance was defined as *P* < 0.05.

## Results

### Clinical efficacy and safety

From 2022 May 9 to 2023 December 31, a total of 83 patients were eligible for enrollment and were included in the ITT population (Fig. 1A and B). The overall study design and workflow are shown in Fig. 1C. Patient baseline characteristics are summarized in Fig. 1D and Table S4, while detailed lesion-level baseline information is provided in Table S5. Representative CT images are presented in Fig. 1E. The distribution of radiotherapy modalities across distinct metastatic organ sites is illustrated in Fig. 1F. The cohort had a median age of 64 years (range: 44 to 75), was predominantly male (84.3%), and had a high smoking prevalence (74.7%). Five patients (6.0%) did not receive Hybrid-RT because of serious complications (*n* = 3, 3.6%) or withdrawal of consent (*n* = 2, 2.4%). Eventually, 78 patients underwent IHBRT without protocol deviation.

**Fig. 1.**
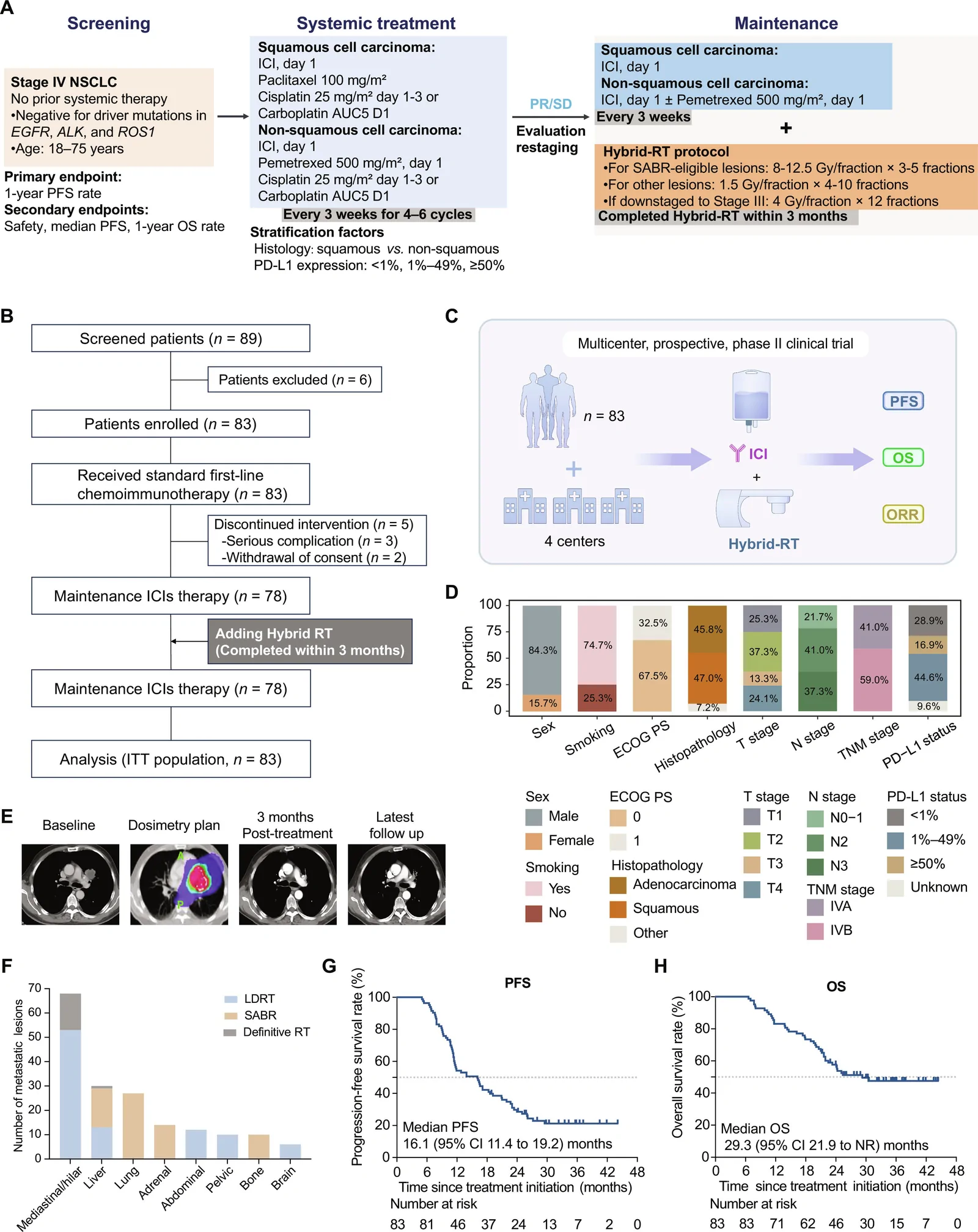
Clinical trial design and survival outcomes. (A) Design and therapeutic schema of the phase II clinical study. (B) Flow diagram of participant enrollment (*n* = 83). (C) Schematic diagram of the multicenter, prospective phase II clinical trial design. (D) Statistical chart of baseline patient characteristics, including sex, smoking, ECOG PS, histopathology, T stage, N stage, TNM stage, and PD-L1 status. (E) Representative CT images of treated patients. (F) Stacked bar chart quantifying lesion counts of different radiotherapy modalities grouped by metastatic sites. (G and H) Kaplan–Meier curves of (G) PFS and (H) OS for participants (*n* = 83). Survival curves were compared using the log-rank test; HRs were estimated using a Cox proportional hazards model. Abbreviations: *ALK*, anaplastic lymphoma kinase; AUC, area under the curve; CI, confidence interval; CT, computed tomography; ECOG PS, Eastern Cooperative Oncology Group performance status; *EGFR*, epidermal growth factor receptor; Hybrid-RT, hybrid dose-fraction radiotherapy; ICI, immune checkpoint inhibitor; ITT, intention to treat; LDRT, low-dose radiotherapy; NR, not reached; NSCLC, non-small cell lung cancer; ORR, objective response rate; OS, overall survival; PD-L1, programmed cell death ligand 1; PFS, progression-free survival; PR, partial response; *ROS1*, ROS proto-oncogene 1; RT, radiotherapy; SABR, stereotactic ablative body radiotherapy; SD, stable disease; TNM, tumor (T)–node (N)–metastasis (M).

At the cutoff date (2025 September 30), the median follow-up for all patients was 24.3 months (interquartile range 6.6 to 44.4 months). In our study, the 1-year PFS rate was 54.2%, exceeding the prespecified target. The 1.5-year and 2-year PFS rates were 42.2% and 28.6%, respectively, with a median PFS of 16.1 months (95% CI 11.4 to 19.2 months; Fig. 1G). The 1-year and 2-year OS rates were 83.1% and 57.7%, respectively, and the median OS was 29.3 months (95% CI 21.9 months to not reached; Fig. 1H). The ORR and DCR were 69.9% (58/83; 95% CI 58.8% to 79.5%) and 91.6% (76/83; 95% CI 83.4% to 96.5%), respectively (Fig. 2A and B). A total of 14 patients (16.9%) were downstaged to stage III and received hypofractionated radiotherapy. Among the remaining 69 patients, median PFS was 16.8 months (95% CI 11.3 to 19.2 months), and median OS was 24.0 months (95% CI 21.2 to 25.4 months). No statistically significant differences in PFS (*P* = 0.57; Fig. S1A) or OS (*P* = 0.45; Fig. S1B) were observed between the 14 patients who were downstaged to stage III and the remaining 69 patients. A total of 32 patients (38.6%) had distant metastasis beyond radiotherapy lesions, and 9 patients (10.8%) experienced both radiotherapy lesion progression and distant metastasis. Additionally, 20 patients (24.1%) experienced progression of LDRT lesions only, whereas no SABR lesions progressed (Table S6)*.*

**Fig. 2.**
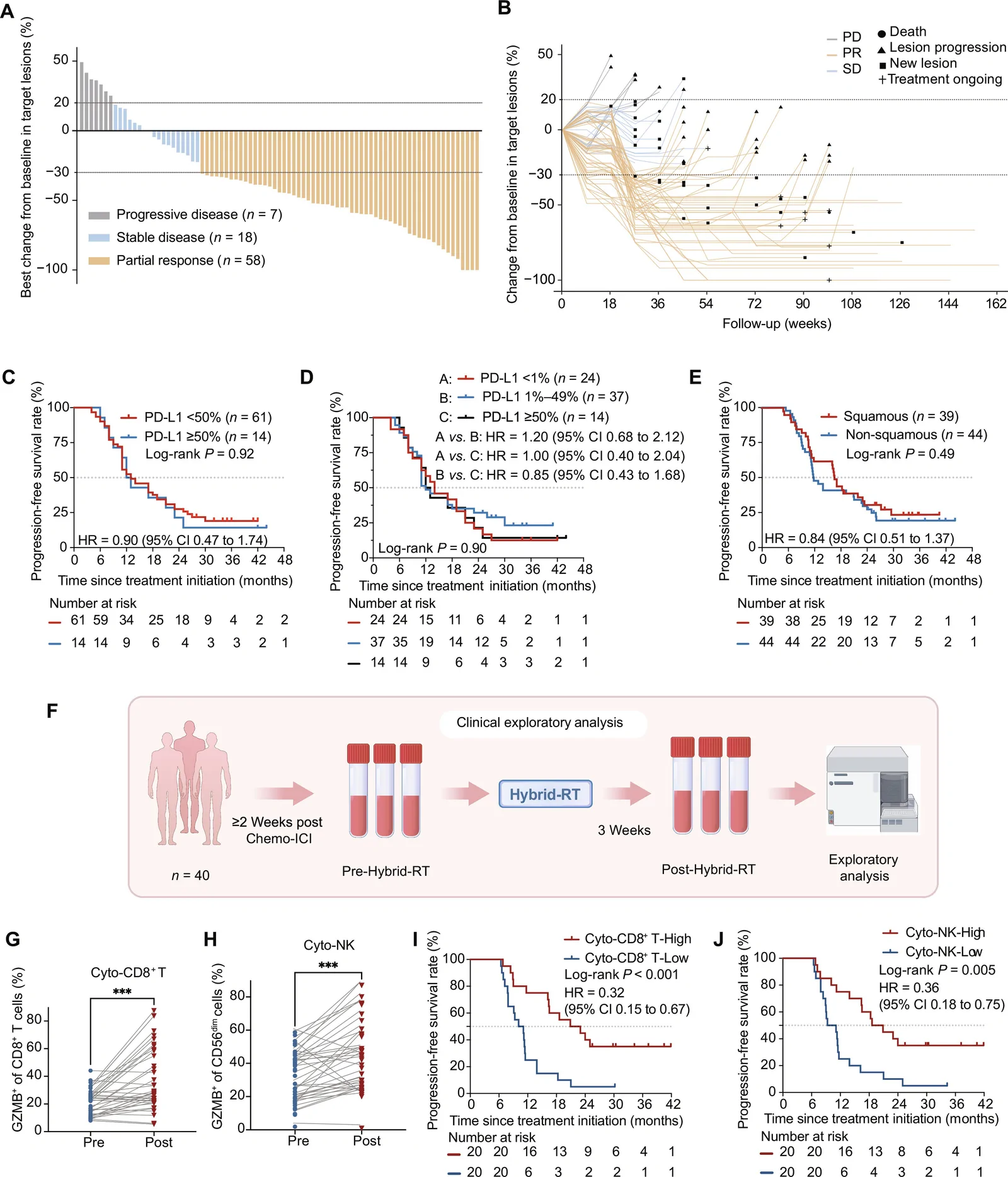
Treatment response and clinical exploratory analysis. (A) Waterfall plot of maximum percentage change in target lesion size from baseline in the ITT population (*n* = 83), evaluated per RECIST v1.1. Each bar represents an individual patient. The horizontal dashed lines indicate a 30% decrease and a 20% increase, corresponding to the thresholds for PR and PD, respectively. SD was defined as a change of >−30% and <20%. (B) Spider plot of target lesion size dynamics from baseline in the ITT population (*n* = 83), evaluated per RECIST v1.1. Each line represents an individual patient. The horizontal dashed lines indicate a 30% decrease and a 20% increase, corresponding to the thresholds for PR and PD, respectively. SD was defined as a change of >−30% and <20%. (C) Kaplan–Meier curves of PFS in PD-L1 TPS ≥50% (*n* = 14) versus <50% (*n* = 61). (D) Kaplan–Meier curves of PFS in PD-L1 subgroups: <1% (*n* = 24), 1% to 49% (*n* = 37), ≥50% (*n* = 14). (E) Kaplan–Meier curves of PFS in squamous (*n* = 39) versus nonsquamous (*n* = 44) carcinoma. (F) Schematic overview of the clinical exploratory analysis workflow (*n* = 40). (G and H) Paired scatter plots of peripheral blood (G) cyto-CD8^+^ T cells defined as CD45^+^CD3^+^CD8^+^CD4^−^GZMB^+^ cells, and (H) cyto-NK cells, defined as CD45^+^CD3^−^CD56^dim^GZMB^+^ cells (*n* = 40). Each dot represents an individual patient; lines connect paired measurements, ***P < 0.001 (paired *t* test). (I and J) Kaplan–Meier curves of PFS stratified by posttreatment ratios of (I) cyto-CD8^+^ cells and (J) cyto-NK cells. *P* values were determined by the log-rank test; HRs were estimated using a Cox proportional hazards model. Abbreviations: Chemo-ICI, chemoimmunotherapy; CI, confidence interval; cyto-CD8^+^, cytotoxic CD8^+^ T cell; cyto-NK, cytotoxic natural killer cell; GZMB, granzyme B; HR, hazard ratio; ICI, immune checkpoint inhibitor; ITT, intention to treat; PD, progressive disease; PD-L1, programmed cell death ligand 1; PFS, progression-free survival; PR, partial response; RECIST, Response Evaluation Criteria in Solid Tumors; RT, radiotherapy; SD, stable disease; TPS, tumor proportion score; vs., versus.

AEs experienced by the study participants are summarized in Table S7. The most common grade 1 to 2 AEs were fatigue (88.0%, 73/83), dermatitis radiation (55.4%, 46/83), lymphocyte count decreased (45.8%, 38/83), neutrophil count decreased (39.8%, 33/83), and nausea and vomiting (34.9%, 29/83). The main grade 3 AEs were lymphocyte count decreased (16.9%, 14/83), aspartate aminotransferase increased (7.2%, 6/83), alanine aminotransferase increased (7.2%, 6/83), and neutrophil count decreased (6.0%, 5/83). In particular, 4 patients (4.8%) experienced grade ≥3 pneumonitis, including 3 patients (3.6%) with grade 3 pneumonitis and 1 patient (1.2%) with grade 4 pneumonitis. No treatment-related deaths occurred. Taken together, these results support the promising clinical efficacy and safety of IHBRT in metastatic NSCLC.

### Subgroup analyses

Next, we evaluated outcomes by PD-L1 status and histology in the ITT population. PD-L1 status was evaluable in 75 patients and not evaluable in the remaining 8 patients, whereas histologic data were available for all 83 patients. The IHBRT regimen showed similar PFS (*P* > 0.05; Fig. 2C to E) and OS (*P* > 0.05; Fig. S1C to E) outcomes across PD-L1 subgroups and histologic subtypes, without evidence of differential efficacy, suggesting that its clinical benefit may be largely independent of baseline PD-L1 expression and histological lineage. To assess whether lesion-based treatment allocation influenced outcomes, we stratified patients by SABR/total lesions (Fig. S1F and G), metastatic burden (Fig. S1H and I), and liver metastasis status (Fig. S1J and K). Stratification showed comparable PFS and OS across subgroups (all *P* > 0.05), indicating efficacy independent of radiotherapy distribution. No significant differences in clinical outcomes were observed among patients who received different ICIs in this study (Fig. S1L and M).

### Clinical exploratory analysis

To explore the potential immunomodulatory effects of Hybrid-RT (initiated ≥2 weeks after chemoimmunotherapy), paired peripheral blood samples were collected before radiotherapy and 3 weeks after radiotherapy from 40 patients and used to perform flow cytometric analysis of lymphocyte subsets (Fig. 2F and Fig. S2A to E), including T cells, NK cells, and other subpopulations. The key findings revealed a significant increase in the proportions of cyto-CD8^+^ T cells and cyto-NK cells after radiotherapy compared with before (both *P* < 0.001; Fig. 2G and H). Using the median value of the proportion of post-radiotherapy cyto-CD8^+^ T cells, we divided the patients into 2 subgroups: cyto-CD8^+^ T-High and cyto-CD8^+^ T-Low. The PFS of cyto-CD8^+^ T-High patients was significantly better than that of cyto-CD8^+^ T-Low patients (median PFS: 22.0 vs. 10.6 months, hazard ratio [HR] = 0.32 [95% CI 0.15 to 0.67]; *P* < 0.001; Fig. 2I). Similarly, compared with cyto-NK-Low patients, cyto-NK-High patients had significantly better PFS (median PFS: 19.8 vs. 10.2 months, HR = 0.36 [95% CI 0.18 to 0.75]; *P* = 0.005; Fig. 2J). These findings suggest that posttreatment enrichment of CD8^+^ T and NK cells, observed following Hybrid-RT during ICI maintenance, correlates with improved clinical outcomes.

### IHBRT exhibits superior antitumor efficacy in NSCLC murine model

Given the observed increase in peripheral cyto-CD8^+^ T and cyto-NK cells following IHBRT and its association with improved PFS, we further investigated whether Hybrid-RT could recapitulate these immune changes in chemotherapy-free preclinical models. We first examined metastatic lesions because they directly receive LDRT, the sole variable that differentiates IHBRT from ISABR (ICI plus SABR). A pilot dose-escalation study was conducted in LLC metastatic models, with mice receiving LDRT at 0, 0.5, 1, or 2 Gy per fraction (total of 3 fractions) to identify the optimal dose for immunomodulation. The 1 Gy/fraction regimen emerged as optimal, eliciting the strongest immune activation in lung metastases by promoting the activation of NK cell-mediated cytotoxicity, antigen processing and presentation, leukocyte transendothelial migration, and chemokine signaling pathways (Fig. S3A).

We then established subcutaneous primary and lung metastatic tumor models using LLC cells, which are reportedly poorly responsive to ICIs [30] and exhibit minimal T cell infiltration [31]. The mice were randomized into 5 groups: untreated control (Control), ICI monotherapy (anti-PD-1), LDRT plus ICI (LDRT + anti-PD-1), ICI plus SABR (ISABR), and IHBRT. While the primary tumors received SABR (8 Gy × 3, BED with an *α*/*β* ratio of 10 Gy [BED_10_] = 43.2 Gy), the metastatic lesions were treated with LDRT (1 Gy × 3, BED_10_ = 3.3 Gy) (Fig. 3A).

**Fig. 3.**
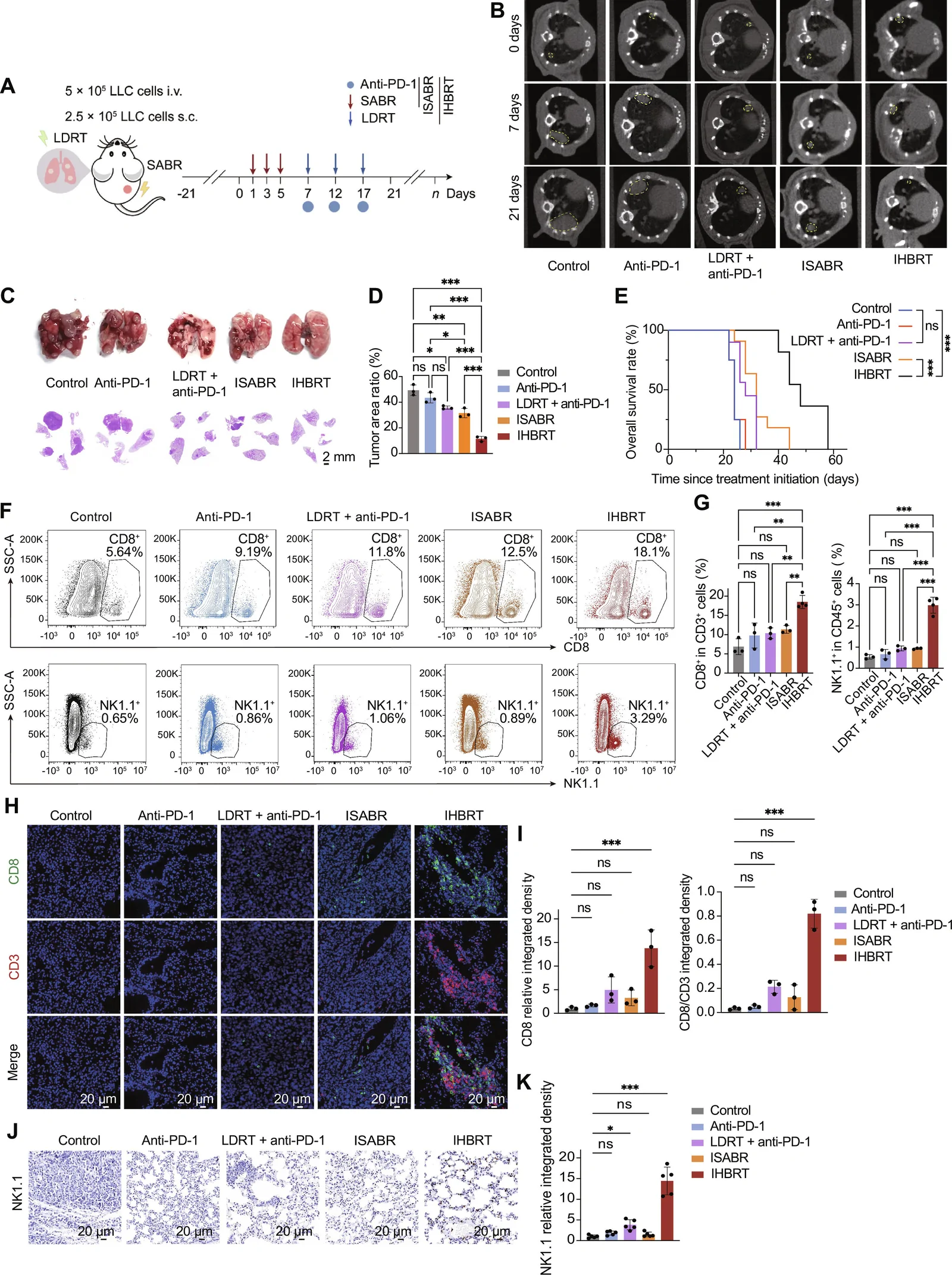
Antitumor efficacy of IHBRT in the NSCLC murine model. (A) Schematic illustration of the in vivo study evaluating different regimens in the NSCLC model. LLC cells were inoculated s.c. and i.v. to establish subcutaneous tumors and lung metastases, respectively. Mice were assigned to the Control, anti-PD-1, LDRT + anti-PD-1, ISABR, or IHBRT groups and received the indicated treatments. (B) Representative lung CT images per treatment group. (C) Representative image (top) and H&E-stained lung sections (bottom) on day 21. (D) Quantified lung tumor area (%) on day 21 (*n* = 3/group). (E) Kaplan–Meier survival curve of NSCLC model mice treated with the indicated groups (*n* ≥ 8). (F) Representative flow cytometry plots of the percentage of CD8^+^ in CD3^+^ (top) and NK1.1^+^ in CD45^+^ cells (bottom) of lung tissue on day 21 (*n* ≥ 3/group). (G) Quantification of the percentage of CD8^+^ in CD3^+^ cells (left) and NK1.1^+^ in CD45^+^ cells (right) of lung tissue on day 21 (*n* ≥ 3/group). (H) Representative mIF images of CD3 (red) and CD8 (green) staining in lung tissue on day 21. (I) Relative integrated density of CD8 (left), and the ratio of CD8/CD3 integrated density (right) in each group (*n* ≥ 3/group). (J) Representative IHC images of NK1.1 staining in lung tissue on day 21. (K) Relative integrated density of NK1.1 staining across indicated groups on day 21 (*n* = 5/group). One-way ANOVA was used for multiple group comparisons. Survival curves were compared using the log-rank test. *P* values are shown, and error bars indicate the mean ± SD. ns, not significant; *P < 0.05; **P < 0.01; ***P < 0.001. Abbreviations: ANOVA, analysis of variance; anti-PD-1, anti-programmed cell death protein 1 antibody; CD3, cluster of differentiation 3; CD8, cluster of differentiation 8; CT, computed tomography; i.v., intravenous; H&E, hematoxylin and eosin; Hybrid-RT, hybrid dose-fraction radiotherapy; ICI, immune checkpoint inhibitor; IHBRT, ICI plus Hybrid-RT; IHC, immunohistochemistry; ISABR, ICI plus SABR; LLC, Lewis lung carcinoma; LDRT, low-dose radiotherapy; mIF, multiplex immunofluorescence; NSCLC, non-small cell lung cancer; s.c., subcutaneous; SABR, stereotactic ablative body radiotherapy; SD, standard deviation.

Compared with the other groups, the IHBRT demonstrated superior control of metastatic tumors and a significant OS advantage. Specifically, compared with ISABR alone, addition of LDRT to the ISABR regimen (forming IHBRT) significantly reduced the metastatic lung tumor area ratio (11.5% vs. 31.5%, *P* < 0.001) and extended the median OS (48 days vs. 32 days, *P* < 0.001) (Fig. 3B to E). Compared with Control, LDRT + anti-PD-1 improved metastasis control (*P* < 0.05); however, the difference in OS between LDRT + anti-PD-1 and Control did not reach statistical significance (median OS 26 vs. 24 days; *P* > 0.05).

To elucidate the contribution of IHBRT to antitumor immunity, we analyzed CD8^+^ T and NK cell infiltration of metastatic lesions and found that compared with ISABR, IHBRT markedly increased CD8^+^ T and NK cell density in metastases (Fig. 3F to K). Notably, ISABR alone modestly reduced metastatic lung area compared with anti-PD-1 alone (*P* < 0.05) and increased CD8^+^ T cell infiltration, suggesting an abscopal effect further amplified by LDRT in IHBRT. All treatment regimens were well tolerated (Fig. S3B). Masson (Fig. S3C) and TGF-β (Fig. S3D) staining revealed comparable collagen and profibrotic signals across all groups. Consistently, GSEA found no group differences in TGF-β signaling and extracellular matrix–receptor interaction pathways between the IHBRT and ISABR groups (Fig. S3E and F). Together with the comparable Masson and TGF-β staining, these findings suggest that supplementary LDRT did not cause evident pulmonary fibrosis in this model. These results indicate that Hybrid-RT enhances the immunogenic tumor microenvironment (TME) and generates synergy between dual radiotherapy modalities to potentiate systemic antitumor immunity, consistent with our clinical observations of improved PFS in NSCLC patients receiving IHBRT.

### IHBRT promotes enrichment of the T/NK subset revealed by single-cell sequencing

To elucidate the immune mechanisms that drive therapeutic synergy, we conducted scRNA-seq of lung tissues from 4 groups: Control, anti-PD-1, ISABR, and IHBRT (Fig. 4A). Unsupervised clustering analysis was performed on the scRNA-seq data obtained from 126,847 quality-controlled cells using uniform manifold approximation and projection (UMAP) to determine the cell-type composition across the experimental groups. Eight clusters were identified (Fig. 4B), and their key marker genes are presented in Fig. 4C. Copy number variation landscapes that are characteristic of malignant transformation were leveraged to demarcate tumor cells from their benign counterparts (Fig. S4A). Gene Ontology (GO) enrichment of differentially expressed genes in the IHBRT group versus the Control group revealed significant enrichment in immune-related pathways in the IHBRT group, including immune system processes, positive regulation of cell migration, the innate immune response, and the major histocompatibility complex (MHC) class I protein complex (Fig. 4D). To better elucidate the immune cell subsets associated with the combination treatment, CD45^+^ tumor-infiltrating immune cells (TIICs) were screened from the 4 groups and subjected to scRNA-seq analysis (Fig. 4E and F). Comparative analysis of immune cell proportions revealed that the IHBRT group exhibited enrichment of T/NK cell subsets (Fig. 4G).

**Fig. 4.**
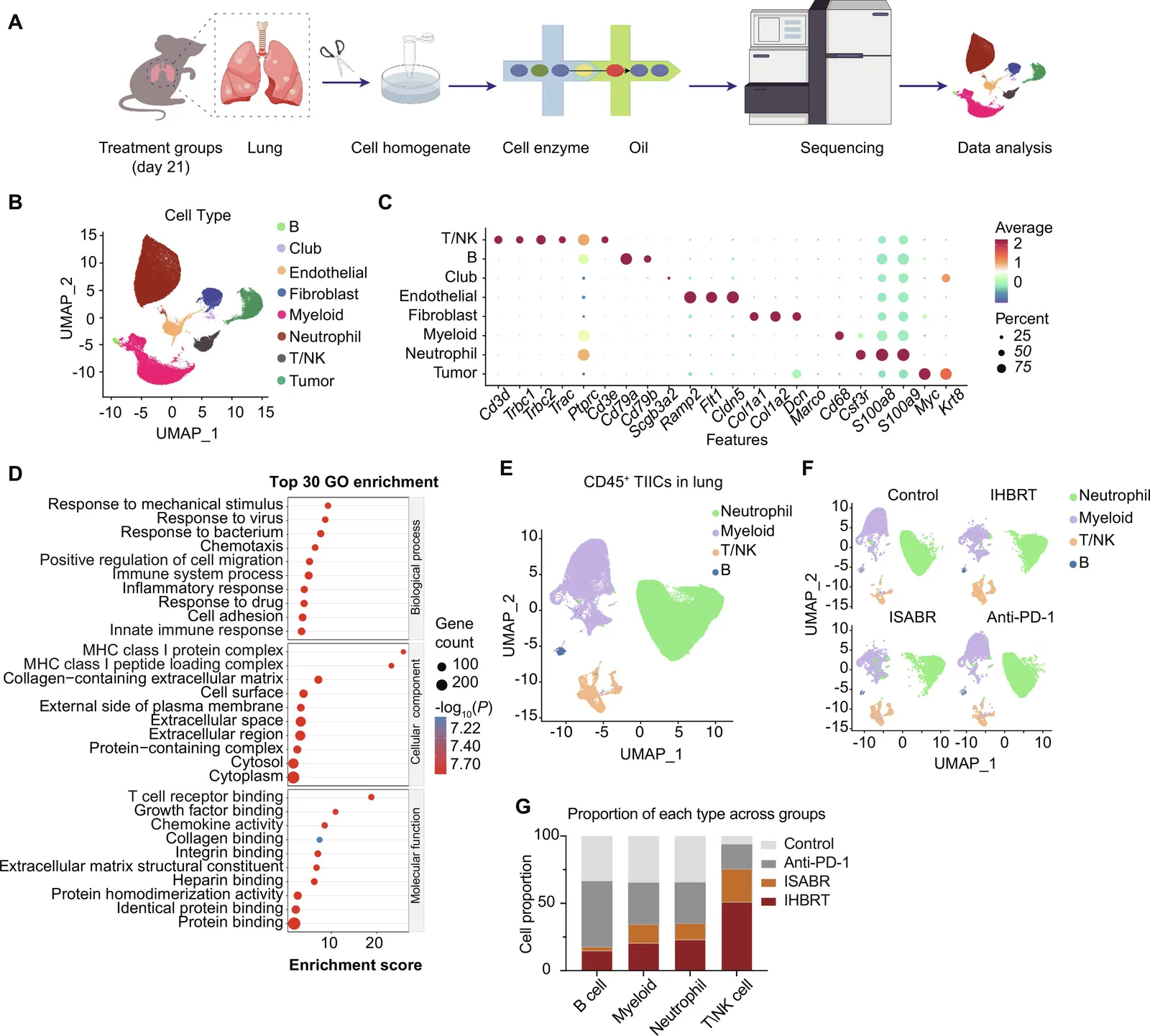
T/NK subset analysis by scRNA-seq. (A) Schematic workflow of scRNA-seq. All tissue samples were harvested on day 21. (B) UMAP plot of scRNA-seq colored by cell type (*n* = 3/group). (C) Average expression and percentage of cells expressing representative marker genes across the indicated cell types. (D) Bubble plot of the top 30 enriched GO terms in the IHBRT group versus the Control group. (E and F) UMAP embeddings of CD45^+^ TIICs, shown for (E) all samples combined and (F) stratified by treatment group. (G) Bar plot showing the proportion of CD45^+^ TIIC subtypes (*n* = 3/group). Differential expression analysis was performed using the Wilcoxon rank-sum test with Benjamini–Hochberg correction. Abbreviations: Anti-PD-1, anti-programmed cell death protein 1 antibody; GO, Gene Ontology; IHBRT, ICI plus Hybrid-RT; ISABR, ICI plus SABR; MHC, major histocompatibility complex; scRNA-seq, single-cell RNA sequencing; TIICs, tumor-infiltrating immune cells; UMAP, uniform manifold approximation and projection.

### IHBRT promotes the infiltration of cyto-CD8^+^ T cells into tumors by activating the Cxcl10–Cxcr3 axis

Subclustering of T/NK cells revealed 9 transcriptionally distinct subtypes: naïve CD8^+^, NK, naïve CD4^+^, cyto-CD8^+^, Th17, cycling T, natural killer T, regulatory T cell (Treg), and type 2 innate lymphoid cells (ILC2) (Fig. 5A and Fig. S4B). Notably, the IHBRT group preferentially increased the proportions of cyto-CD8^+^ and NK subsets compared to other groups (Fig. 5B**)**. Given that the impact of LDRT combined with ISABR remains unclear, we specifically compared the immune effects of IHBRT to ISABR alone. Cell–cell communication analysis between T/NK and tumor cells demonstrated that IHBRT increased both the number of ligand–receptor pairs and their interaction strength compared with ISABR (Fig. 5C to E and Fig. S4C and D). CellChat analysis revealed that C-C motif chemokine ligand (CCL) and C-X-C motif chemokine ligand (CXCL) signaling pathways exhibited the highest information flow between cyto-CD8^+^ T cells and tumor cells (Fig. 5F and G). Among all ligand receptor pairs, the *Cxcl10–Cxcr3* interaction from tumor cells to cyto-CD8^+^ T cells showed a marked increase in communication probability in IHBRT versus ISABR (Fig. S4E), and this increase was largely confined to this cell–pair interaction. Although *Ccl2–Ccr2* showed a larger increase, this change was not specific to the tumor cell and cyto-CD8^+^ T cells and was also observed in macrophages; we therefore focused on the *Cxcl10–Cxcr3* interaction. *Cxcl10* was predominantly expressed by tumor cells, with minimal contribution from other cell types (Fig. S4F). This was further supported by increased *Cxcl10* expression in tumor cells and elevated *Cxcr3* in cyto-CD8^+^ T cells (Fig. 5H). Flow cytometry confirmed that IHBRT markedly increased Cxcr3 expression intensity of cyto-CD8^+^ T cells compared to other groups (Fig. 5I and J). Consistent with these findings, serum levels of Cxcl10 and Cxcl11, both of which are Cxcr3 ligands [32], were elevated in IHBRT-treated mice (Fig. 5K). In addition, mIF staining confirmed increased Cxcl10 expression in the TME and elevated Cxcr3 expression on infiltrating cyto-CD8^+^ cells (Fig. 5L).

**Fig. 5.**
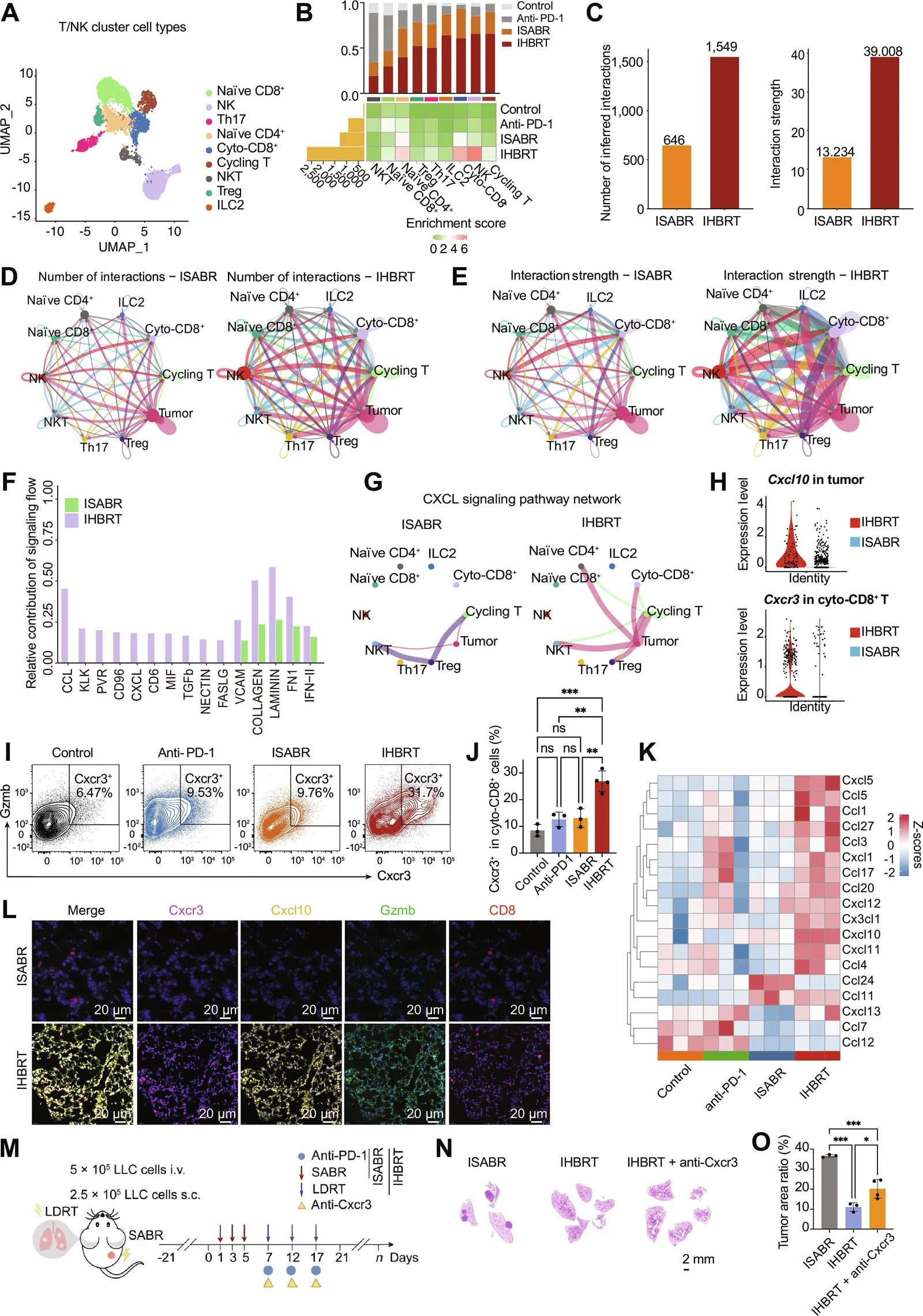
Cxcl10–Cxcr3 axis and CD8^+^ T cell infiltration analysis. (A) UMAP plot of T/NK-subtype cells from scRNA-seq data colored by cell type (*n* = 3/group). (B) T/NK subtype distribution: proportions (top), enrichment (heatmap), and total counts (bar plot) (*n* = 3/group). (C) Number of ligand–receptor pairs (left) and interaction strength (right) between T/NK subtype and tumor cells (*n* = 3/group). (D and E) Cell–cell interaction networks among T/NK subtype and tumor cells in the ISABR and IHBRT groups, inferred from scRNA-seq data of lung metastatic lesions (*n* = 3/group). Nodes represent cell populations. Edge thickness represents (D) the number of inferred interactions and (E) interaction strength. (F) Bar plot showing the relative contribution of signaling flow between tumor and cyto-CD8^+^ T cells via differential ligand–receptor pairs (ISABR vs. IHBRT). (G) CXCL signaling networks between T/NK subtype and tumor cells (*n* = 3/group). (H) Violin plot showing *Cxcl10* expression in tumors (upper) and *Cxcr3* expression in cyto-CD8^+^ T cells (lower). (I and J) Representative (I) flow cytometry plots and (J) percentage quantification of Cxcr3^+^ in cyto-CD8^+^ cells of lung tissue on day 21 (*n* ≥ 3/group). (K) Heatmap of serum chemokine levels in the treatment groups, measured by Luminex multiplex assay (*n* = 3/group). (L) Representative mIF images of lung tissue from the ISABR and IHBRT groups on day 21. (M to O) Cxcr3 blockade study design. (M) Schematic diagram of the experimental procedure. LLC cells were inoculated s.c. and i.v. to establish subcutaneous tumors and lung metastases, respectively. Mice were assigned to the ISABR, IHBRT, or IHBRT plus anti-Cxcr3 groups and received the indicated treatment regimens. (N) Representative H&E-stained lung sections on day 21. (O) Quantified lung tumor area ratio (%) on day 21 (*n* ≥ 3/group). Comparisons between 2 groups were performed using the unpaired Student’s *t* test; comparisons among multiple groups were performed using one-way ANOVA with Tukey’s post hoc test. Error bars indicate mean ± SD. ns, not significant; *P < 0.05; **P < 0.01; ***P < 0.001. Abbreviations: ANOVA, analysis of variance; anti-Cxcr3, anti-C-X-C motif chemokine receptor 3 antibody; anti-PD-1, anti-programmed cell death protein 1 antibody; CCL, C-C motif chemokine ligand; CD6, cluster of differentiation 6; CD8, cluster of differentiation 8; CD96, cluster of differentiation 96; COLLAGEN, collagen family; Cx3cl, C-X3-C motif chemokine ligand 1; CXCL, C-X-C motif chemokine ligand; Cxcr3, C-X-C motif chemokine receptor 3; cyto-CD8^+^, cytotoxic CD8^+^ T cell; FASLG, Fas ligand; FN1, fibronectin 1; Gzmb, granzyme B; H&E, hematoxylin and eosin; IFN-II, type II interferon; IHBRT, ICI plus Hybrid-RT; ILC2, innate lymphoid cell type 2; i.v., intravenous; ISABR, ICI plus SABR; KLK, kallikrein-related peptidase; LLC, Lewis lung carcinoma; MIF, macrophage migration inhibitory factor; mIF, multiplex immunofluorescence; NECTIN, nectin family; NKT, natural killer T cell; PVR, poliovirus receptor; s.c., subcutaneous; SABR, stereotactic ablative body radiotherapy; scRNA-seq, single-cell RNA sequencing; SD, standard deviation; TGF-β, transforming growth factor beta; Th17, T helper 17 cell; Treg, regulatory T cell; UMAP, uniform manifold approximation and projection; VCAM, vascular cell adhesion molecule; vs., versus.

Functionally, Cxcr3 blockade markedly attenuated IHBRT efficacy, with metastatic lung area increasing from 11.01% ± 2.18% (IHBRT) to 20.18% ± 4.72% (IHBRT + anti-Cxcr3, *P* < 0.05, Fig. 5M to O). Of note, tumor burden after Cxcr3 blockade remained lower than that of ISABR alone (20.18% vs. 36.64%, *P* < 0.001), indicating that while the Cxcl10–Cxcr3 axis contributes to IHBRT-driven metastasis control, additional mechanisms may contribute. Collectively, these results demonstrate that IHBRT amplifies the Cxcl10–Cxcr3-mediated recruitment of cyto-CD8^+^ cells into tumors, and further identifies Cxcr3 as a key mechanistic driver underlying IHBRT-mediated metastatic lung cancer control, while also hinting at complementary pathways that may synergize to mediate the therapeutic effects of IHBRT.

### IHBRT enhances NK cell antitumor activity through up-regulation of NKG2D

We next examined whether IHBRT also engages innate immunity, particularly NK cells. Kyoto Encyclopedia of Genes and Genomes (KEGG) pathway analysis of T/NK cells demonstrated significant enrichment in the NK cell-mediated cytotoxicity pathway in the IHBRT group relative to the ISABR group (Fig. 6A). Accordingly, the activating receptor NKG2D (*Klrk1*) was up-regulated in NK cells from IHBRT-treated mice (Fig. 6B), and flow cytometry confirmed a higher frequency of NKG2D^+^ NK cells in lung tissues (Fig. 6C and D). Given that NKG2D recognizes stress-induced ligands such as NKG2D ligands (NKG2DL) that are expressed on tumor cells [33], IHBRT-treated metastases were further characterized by the concomitant up-regulation of NKG2D in NK cells and NKG2DL within the TME (Fig. 6E).

**Fig. 6.**
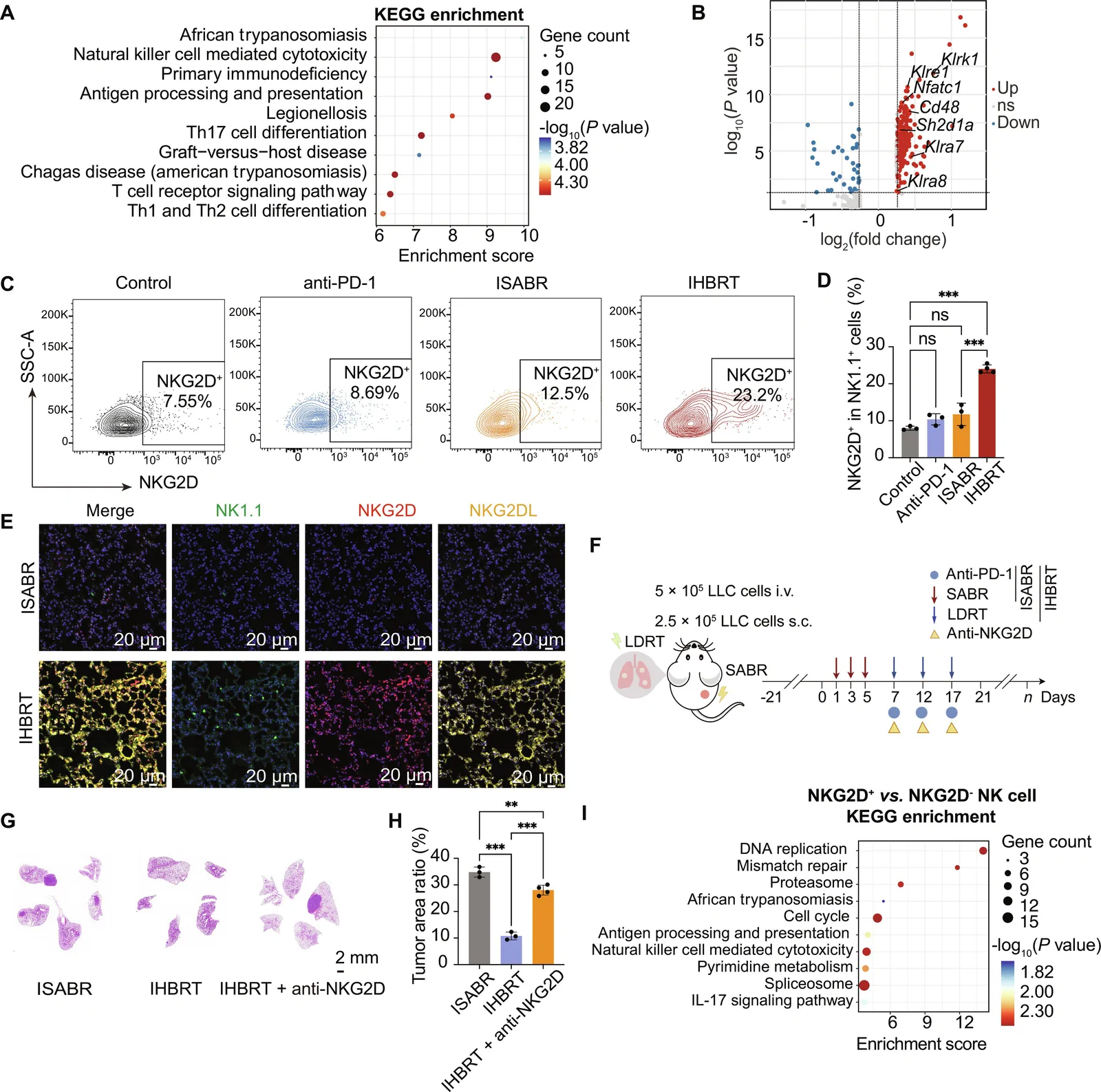
NK-cell antitumor activity and NKG2D signaling analysis. (A) Bubble plot showing enriched KEGG pathways of T/NK subtype in IHBRT versus ISABR. (B) Volcano plot showing differentially expressed genes of the NK subset between IHBRT and ISABR. Red: significantly up-regulated genes, *P* < 0.05 and log_2_(fold change) > 0.25. Blue: significantly down-regulated genes, *P* < 0.05 and log_2_(fold change) < −0.25. Gray: ns. (C and D) Representative (C) flow cytometry plots and the (D) proportion of NKG2D^+^ NK1.1^+^ cells in lung tissue (*n* ≥ 3/group). (E) Representative mIF images of lung tissue on day 21. (F) NKG2D blockade study design. LLC cells were inoculated s.c. and i.v. to establish subcutaneous tumors and lung metastases, respectively. Mice were assigned to the ISABR, IHBRT, or IHBRT + anti-NKG2D groups and received the indicated treatment regimens to assess the effect of NKG2D blockade. (G) Representative H&E-stained lung sections on day 21. (H) Quantified lung tumor area (%) on day 21 (*n* ≥ 3/group). (I) Bubble plot showing the top 10 enriched KEGG pathways in NKG2D^+^ versus NKG2D^−^ NK cells. Differential expression analysis was performed using the Wilcoxon rank-sum test with Benjamini–Hochberg correction. Comparisons between 2 groups were performed using the unpaired Student’s *t* test; comparisons among multiple groups were performed using one-way ANOVA with Tukey’s post hoc test. Error bars indicate mean ± SD. ns, not significant; *P < 0.05; **P < 0.01; ***P < 0.001. Abbreviations: ANOVA, analysis of variance; anti-NKG2D, anti-NKG2D antibody; anti-PD-1, anti-programmed cell death protein 1 antibody; CD48, cluster of differentiation 48; H&E, hematoxylin and eosin; i.v., intravenous; IHBRT, ICI plus Hybrid-RT; IL-17, interleukin-17; ISABR, ICI plus SABR; KEGG, Kyoto Encyclopedia of Genes and Genomes; *Klra*, killer cell lectin-like receptor subfamily A member; *Klre1*, killer cell lectin-like receptor subfamily E member 1; *Klrk1*, killer cell lectin-like receptor subfamily K member 1; LDRT, low-dose radiotherapy; LLC, Lewis lung carcinoma; mIF, multiplex immunofluorescence; NK, natural killer; NKG2D, natural killer group 2 member D; *Nfatc1*, nuclear factor of activated T cells 1; NKG2DL, NKG2D ligand; SABR, stereotactic ablative body radiotherapy; s.c., subcutaneous; *Sh2d1a*, SH2 domain containing 1A; SD, standard deviation; Th, T helper.

To assess functional relevance, we performed NKG2D blockade (Fig. 6F). Similar to Cxcr3 disruption, NKG2D blockade significantly attenuated IHBRT efficacy against metastatic disease: lung tumor area increased from 10.79% ± 1.47% (IHBRT) to 28.05% ± 1.86% (IHBRT + anti-NKG2D, *P* < 0.001), whereas the lung tumor area was 34.82% ± 1.92% with ISABR alone (Fig. 6G and H). Together with the Cxcr3 blockade results, these findings indicate that both Cxcr3 and NKG2D contribute to IHBRT-mediated control of pulmonary metastases. Transcriptomic comparisons of NKG2D^+^ and NKG2D^-^ NK cells revealed significant enrichment in cell cycle-related genes and DNA replication-related genes in the NK subset (Fig. 6I). Thus, NKG2D up-regulation contributes functionally to IHBRT-mediated antitumor activity against metastatic NSCLC.

### IHBRT suppresses Spp1^+^ macrophages and disrupts macrophages–fibroblast interactions in metastatic lesions

Building on the immune activation induced by IHBRT, we next examined the myeloid cells in metastatic lesions and clarified the mechanisms by which LDRT attenuates immunosuppression. Analysis of myeloid subsets (Fig. 7A and B), which mainly identified monocytes, macrophages, and DCs, revealed that the addition of LDRT to ISABR (forming IHBRT) had no marked effect on the proportion of each subset (Fig. 7C). Spp1^+^ macrophages have an established role in promoting immunosuppressive fibroblast barriers that drive tumor progression [34–36]. Notably, CellChat analysis revealed that IHBRT markedly attenuated Spp1-mediated interactions between macrophages and fibroblasts compared with ISABR (Fig. 7D and E). Consistently, *Spp1* expression in macrophages was significantly reduced in the IHBRT group compared with ISABR (Fig. 7F). A high SPP1*^+^* macrophage signature score was associated with poorer OS in the combined LUAD and LUSC (*P* = 0.029; Fig. 7G), underscoring the clinical relevance of SPP1^+^ macrophages. Flow cytometry (Fig. 7H) and mIF (Fig. 7I) confirmed reduced Spp1^+^ macrophage density in IHBRT-treated metastatic lesions. Overall, IHBRT disrupts Spp1^+^ macrophage–fibroblast interactions and remodels the metastatic microenvironment to facilitate lymphocyte infiltration and antitumor immunity.

**Fig. 7.**
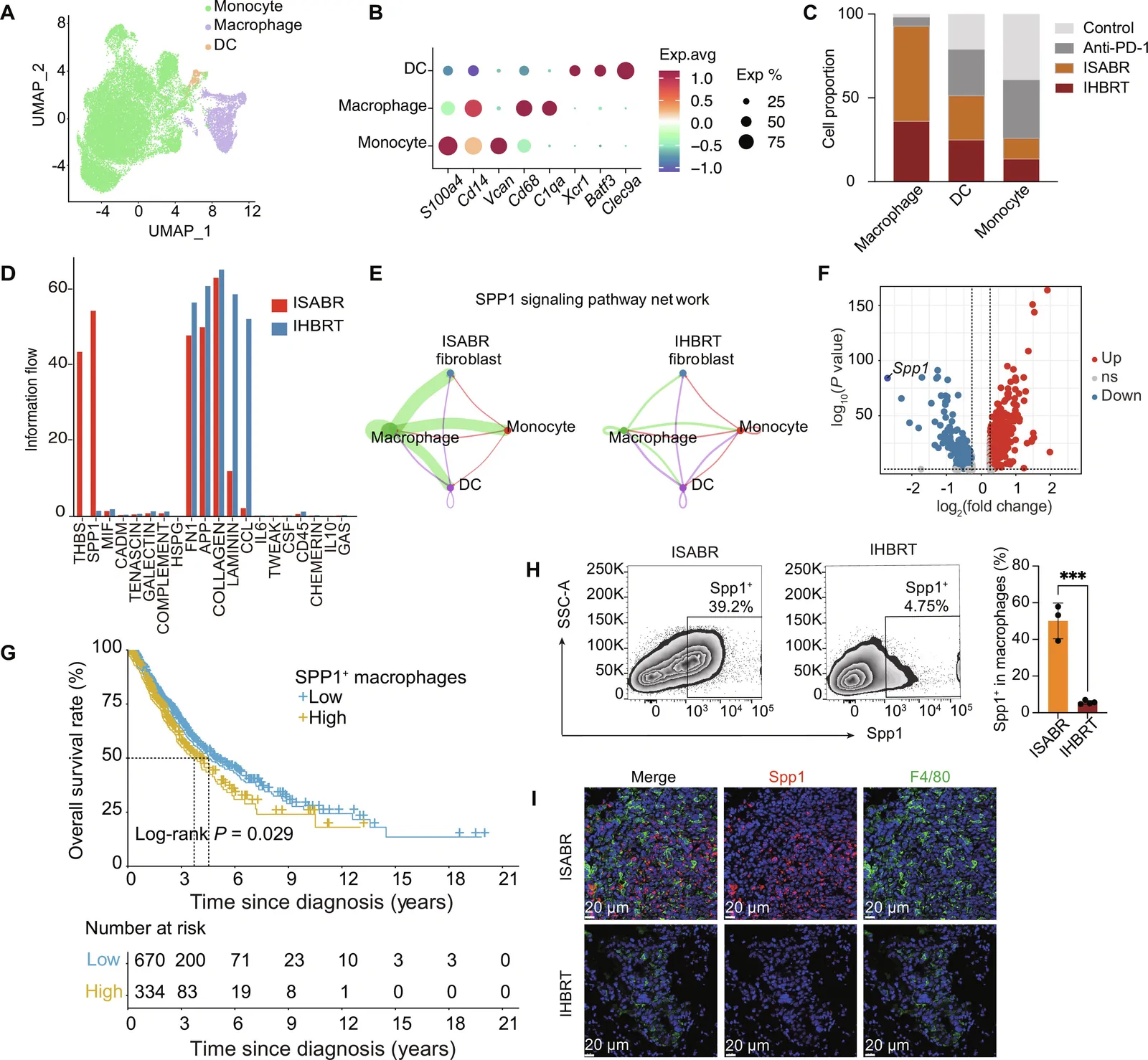
Myeloid–fibroblast interaction and Spp1^+^ macrophage analysis in metastatic lesions. (A) UMAP plot of myeloid-subtype cells from scRNA-seq data of lung tissues from the Control, anti-PD-1, ISABR, and IHBRT groups (*n* = 3/group), colored by cell type. (B) Average expression and percentage of cells expressing representative marker genes across the indicated myeloid-subtype cells. (C) Bar plot showing the relative proportion of cells from 4 treatment groups for myeloid subtypes (*n* = 3/group). (D) Information flow showing signaling pathways ranked based on differences between ISABR and IHBRT. (E) Communication of the SPP1 signaling pathway network between myeloid subtypes and fibroblast cells. (F) Volcano plot showing differentially expressed genes of macrophages between IHBRT and ISABR. Red: significantly up-regulated genes, *P* < 0.05 and log_2_(fold change) > 0.25. Blue: significantly down-regulated genes, *P* < 0.05 and log_2_(fold change) < −0.25. Gray: ns. (G) Kaplan–Meier curves of OS in TCGA-LUAD and TCGA-LUSC cohorts. Patients were dichotomized by the SPP1^+^ macrophage signature score at the data-driven optimal cutoff (high score, *n* = 334; low score, *n* = 670). (H) Representative flow cytometry plots (left) and the proportion (right) of Spp1^+^ in CD11b^+^ F4/80^+^ cells in lung tissue (*n* ≥ 3/group). (I) Representative mIF images in lung tissue on day 21. Differential expression analysis was performed using the Wilcoxon rank-sum test with Benjamini–Hochberg correction. Survival curves were compared using the log-rank test; HRs were estimated using a Cox proportional hazards model. Error bars indicate mean ± SD. ns, not significant; *P < 0.05; **P < 0.01; ***P < 0.001. Abbreviations: ANOVA, analysis of variance; anti-PD-1, anti-programmed cell death protein 1 antibody; CD11b, cluster of differentiation 11b; DC, dendritic cell; IHBRT, ICI plus Hybrid-RT; ISABR, ICI plus SABR; KEGG, Kyoto Encyclopedia of Genes and Genomes; mIF, multiplex immunofluorescence; OS, overall survival; scRNA-seq, single-cell RNA sequencing; SD, standard deviation; SPP1, secreted phosphoprotein 1; TCGA-LUAD, The Cancer Genome Atlas-lung adenocarcinoma; TCGA-LUSC, The Cancer Genome Atlas-lung squamous cell carcinoma; UMAP, uniform manifold approximation and projection; ns, not significant.

### Systemic immune activation by IHBRT potentiates the ablative effects of SABR lesions

We next examined primary tumors to determine whether IHBRT-induced immune activation in metastases elicits systemic antitumor response. Despite the absence of direct delivery of LDRT to primary tumors (Fig. 8A), IHBRT markedly enhanced primary tumor regression compared with ISABR. Strikingly, tumor burden was significantly higher after Cxcr3 blockade than after NKG2D blockade (1,896 mm^3^ vs. 1,111 mm^3^, *P* < 0.001), positioning Cxcr3-mediated CD8^+^ T cells as the dominant drivers of primary tumor regression, with only a minor contribution from NKG2D-mediated NK cells (Fig. 8B to D). Moreover, mIF and flow cytometry analyses revealed pronounced IHBRT-induced accumulation of intratumoral CD8^+^ T cells (Fig. 8E to G). Compared with ISABR, IHBRT also increased serum levels of T cell-related cytokines, including interferon-gamma (IFN-γ), granulocyte-macrophage colony-stimulating factor (GM-CSF), interleukin-2 (IL-2), and tumor necrosis factor-alpha (TNF-α) (*P* < 0.05 for all; Fig. 8H to K), indicating systemic immune activation. To assess systemic antitumor immunity, we additionally implanted a secondary tumor on the contralateral flank (Fig. 8L). Consistent with a systemic response, contralateral tumors that did not receive radiation displayed significantly greater regression in the IHBRT group than in the ISABR group (*P* < 0.001, Fig. 8M to O). Thus, while NKG2D-armed NK cells contribute to the control of pulmonary metastases, Cxcr3*-*mediated CD8^+^ T cell infiltration serves as the dominant effector axis within the primary tumor. This division of labor enables IHBRT to simultaneously control both local and metastatic disease, as summarized in the mechanistic schematic (Fig. 8P).

**Fig. 8.**
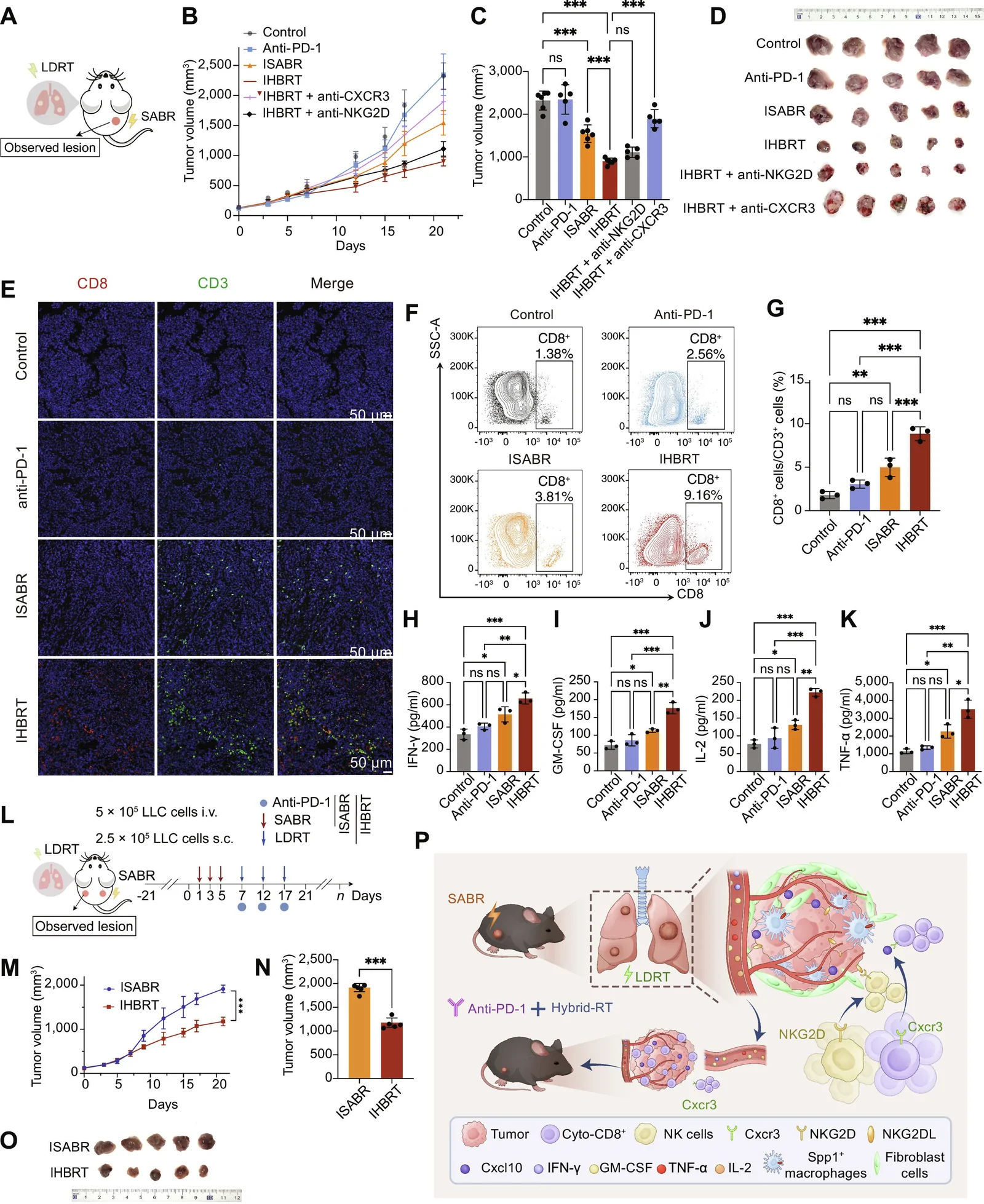
Systemic immune activation and abscopal antitumor effects of IHBRT. (A) Schematic diagram of the in vivo treatment regimen targeting the observed subcutaneous tumors. (B) Tumor growth curves at SABR sites in the Control, anti-PD-1, ISABR, IHBRT, IHBRT + anti-NKG2D, and IHBRT + anti-Cxcr3 groups. (C and D) Tumor (C) volume and (D) representative images of subcutaneous tumors on day 21. (E) Representative mIF image of CD8 and CD3 in tumor tissue across the indicated groups. (F and G) Representative (F) flow cytometry plots and (G) percentage quantification of CD8^+^ cells among CD3^+^ cells of tumor tissue TIICs (*n* ≥ 3/group). (H to K) Bar plots showing serum levels of (H) IFN-γ, (I) GM-CSF, (J) IL-2, and (K) TNF-α, measured by Luminex assay (*n* = 3/group). (L) Schematic illustration of the secondary tumor study design. LLC cells were inoculated s.c. bilaterally and i.v. to establish bilateral subcutaneous tumors and lung metastases, respectively. Mice were assigned to the ISABR and IHBRT groups and received the indicated treatments. SABR was delivered to one subcutaneous tumor in the ISABR and IHBRT groups, and growth of the contralateral, nonirradiated tumor was monitored to assess systemic antitumor immunity. (M) Secondary tumor growth curves (*n* = 5/group). (N and O) Secondary tumor (N) volume and (O) representative images on day 21. (P) Schematic overview of the proposed mechanism underlying IHBRT-mediated antitumor immunity. Schematic illustration created with FigDraw. Comparisons between 2 groups were performed using the unpaired Student’s *t* test; comparisons among multiple groups were performed using one-way ANOVA with Tukey’s post hoc test. Error bars indicate mean ± SD. ns, not significant; *P < 0.05; **P < 0.01; ***P < 0.001. Abbreviations: ANOVA, analysis of variance; anti-Cxcr3, anti-C-X-C motif chemokine receptor 3 antibody; anti-NKG2D, anti-NKG2D antibody; anti-PD-1, anti-programmed cell death protein 1 antibody; CD3, cluster of differentiation 3; CD8, cluster of differentiation 8; Cxcl10, C-X-C motif chemokine ligand 10; Cxcr3, C-X-C motif chemokine receptor 3; GM-CSF, granulocyte-macrophage colony-stimulating factor; Hybrid-RT, hybrid dose-fraction radiotherapy; IFN-γ, interferon-γ; IHBRT, ICI plus Hybrid-RT; IL-2, interleukin-2; i.v., intravenous; ISABR, ICI plus SABR; LDRT, low-dose radiotherapy; LLC, Lewis lung carcinoma; mIF, multiplex immunofluorescence; NKG2D, natural killer group 2 member D; NKG2DL, NKG2D ligand; SABR, stereotactic ablative body radiotherapy; s.c., subcutaneous; SD, standard deviation; Spp1, secreted phosphoprotein 1; TIICs, tumor-infiltrating immune cells; TNF-α, tumor necrosis factor-α.

## Discussion

This prospective, multicenter phase II study evaluated the efficacy and safety of ICI combined with Hybrid-RT in patients with metastatic driver gene-negative NSCLC. Hybrid-RT is selected based on individual lesions. It adopts a dose-fractionation regimen characterized by short-course, low-total-dose radiotherapy. This approach theoretically reduces toxicity to organs at risk and minimizes radiation-induced immunosuppression, making it more suitable for combination with ICI therapy [6,37,38]. The study met its primary endpoint, with a 1-year PFS rate of 54.2% and a median PFS of 16.1 months, representing clinically meaningful and highly promising outcomes.

In this trial, radiotherapy was administered during ICI maintenance after chemoimmunotherapy. Platinum-based chemoimmunotherapy achieves maximal tumor response in the induction phase but carries substantial toxicity. Thus, most novel ICI combinations are increasingly investigated in the maintenance setting [39–41]. Our sequential design was rationally constructed to avoid additive toxicity from concurrent chemo-immunoradiotherapy and minimize target volumes by delivering focal radiotherapy in the setting of reduced tumor burden. This optimized treatment sequence appears to account for both the promising survival efficacy and the favorable safety outcomes.

Based on the “in situ tumor vaccine” concept, SABR plus ICI has been widely explored in advanced solid tumors. Although the clinical benefit of ISABR has been demonstrated in NSCLC, the reported outcomes show considerable heterogeneity across studies [11,42,43]. This heterogeneity is largely driven by inter- and intratumoral genomic diversity, which generates distinct immune microenvironments in primary and metastatic sites [44,45], limiting systemic immune activation and consistent abscopal effects [46]. To address this problem, some clinicians have proposed irradiating multiple lesions rather than relying on single-site SABR [47]. However, that approach is often limited by technical challenges, including the inability to deliver ablative doses to large tumors and to tumors that are located close to critical structures [48]. The local efficacy of SABR in this trial may also reflect the intrinsic potency of ablative doses rather than immune effects alone. Accordingly, LDRT has emerged as a practical complementary strategy to remodel the TIME [21,49,50], enhance immune cell infiltration, and potentiate antitumor immunity [49,51].

Given the complementary immunomodulatory effects of SABR and LDRT, the triple combination of SABR + LDRT + ICI is biologically rational [22,52,53]. A phase Ib study evaluated a chemotherapy-free regimen that combines SABR, LDRT, and sintilimab for the treatment of PD-L1-positive stage IV NSCLC and revealed acceptable safety and evidence of LDRT-induced immune modulation [24]. However, its median PFS of 8.6 months did not exceed historical benchmarks for standard ICI or chemoimmunotherapy [54,55]. By contrast, our study used standard first-line chemoimmunotherapy regardless of PD-L1 status. Although PD-L1-high tumors may derive greater benefit from single-agent ICI, chemotherapy-free strategies remain restricted to patients with PD-L1 ≥50% [56,57]. Importantly, our results revealed no significant PFS or OS advantage in PD-L1-high patients compared with PD-L1-low/negative patients. IHBRT triggers broad immune activation and reshapes the tumor immune landscape, with efficacy observed across PD-L1 subgroups. This is consistent with trials suggesting radiotherapy may benefit PD-L1-negative/low NSCLC [11,58,59], supporting its potential broader applicability. We acknowledge that the lack of posttreatment biopsies prevents us from evaluating dynamic PD-L1 alterations during treatment. Additionally, no significant difference in PFS and OS was observed between squamous and nonsquamous subgroups in this study, despite prior reports suggesting squamous NSCLC may have inferior chemoimmunotherapy responses [28,60–62]. Thus, IHBRT demonstrates broad applicability irrespective of PD-L1 expression and histologic subtype.

The results of our exploratory clinical study demonstrated that compared with preradiotherapy levels, the proportions of cyto-CD8^+^ T cells and cyto-NK cells increased significantly after IHBRT. We further explored the underlying mechanisms using a murine metastatic NSCLC model. Previous studies have shown that LDRT combined with immunotherapy can convert the TIME into one characterized by extensive infiltration by CD8^+^ T cells, particularly exhausted CD8^+^ T (CD8^+^ T_ex_) cells [21,63,64]. LDRT combined with anti-PD-1 treatment prolonged OS in the NSCLC metastatic model, although median OS did not differ statistically between the Control and anti-PD-1 groups. This may be due to the limited release of tumor antigens [65], which would impair effective tumor control. By contrast, IHBRT induced a “hot” immune phenotype in LDRT lesions and enriched functionally activated effector CD8^+^ T cells via the Cxcl10–Cxcr3 axis, rather than exhausted T cells. Notably, ISABR alone induced abscopal effects in lung metastases, which were substantially augmented by adding LDRT to metastatic sites. The robust serum cytokine elevation induced by IHBRT, but not by ISABR alone, aligns with the enhanced systemic antitumor responses observed in contralateral tumors, supporting that LDRT amplifies systemic immunity beyond SABR alone. These results indicate that SABR-mediated sustained antigen release synergizes with LDRT-mediated immune priming to generate robust systemic immunity.

Moreover, our in vivo studies demonstrated that IHBRT up-regulated NKG2D on NK cells and NKG2DL on tumor cells, enhancing NK cell cytotoxicity by modulating DNA replication and cell cycle progression. These findings are consistent with prior reports that radiation up-regulates NKG2DL in tumor cells, thereby potentiating NKG2D-targeted immunotherapy [66]. Importantly, our results delineate a spatial division of labor between CD8^+^ T cells and NK cells under IHBRT: Cxcr3-mediated CD8^+^ T cells contribute to primary tumor control, whereas NKG2D-mediated NK cells contribute to the regression of metastatic lesions, with coordinated support from Cxcl10–Cxcr3-mediated CD8^+^ T cells. This immune effector program enables IHBRT to exert robust local control and coordinated systemic activity against distant metastases.

In addition to direct effector activation, our in vivo findings showed that IHBRT remodeled the suppressive myeloid compartment by suppressing Spp1^+^ macrophages and disrupting Spp1-dependent crosstalk with fibroblasts. Given the role of Spp1^+^ macrophages in promoting immunosuppressive fibroblast barriers [34–36], these effects likely contribute to the dismantling of these barriers. Together, these findings provide a novel mechanistic foundation for radiotherapy–immunotherapy synergy and fill a critical mechanistic gap for combined SABR + LDRT strategies.

While our study provides promising mechanistic insights and demonstrates therapeutic potential, it has some limitations that should be noted. First, the single-arm design and modest sample size may limit generalizability. Second, the scarcity of posttreatment biopsy specimens limited our ability to comprehensively assess dynamic changes in immune cell subsets. Third, although no significant differences in clinical outcomes were observed among patients who received different ICIs in this study, heterogeneous ICI selection without protocol standardization introduces confounding variables. In addition, in our radiotherapy protocol, the general framework combines LDRT with SABR. However, specific dosing regimens remain individualized across patients. Subgroup analyses by metastatic burden, SABR coverage, and liver metastasis status, together with an analysis excluding patients downstaged to stage III, yielded broadly consistent findings. Nevertheless, future randomized controlled trials with expanded cohorts are needed to standardize the radiotherapy protocol and minimize bias. Finally, although murine models offer valuable preclinical insights, they incompletely mimic the complexity of human TIMEs. Findings from these preclinical models therefore require validation in future clinical studies.

Building on the compelling clinical and mechanistic evidence for IHBRT in driver-negative metastatic NSCLC presented in this study, we suggest that multicenter randomized phase III trials represent the critical next step in definitively establishing both the therapeutic efficacy and the safety profile of this combined approach.

## Conclusions

In conclusion, our study demonstrates that IHBRT yields an encouraging PFS and a manageable safety profile in patients with driver-negative metastatic NSCLC. Mechanistically, our in vivo studies revealed that IHBRT could induce a functional division of labor: Cxcr3-mediated CD8^+^ T cells dominate primary tumor control, while NKG2D-mediated NK cells and Cxcr3-mediated CD8^+^ T cells contribute to metastatic lesion regression. Concurrently, IHBRT suppresses Spp1^+^ macrophages and disrupts their crosstalk with fibroblasts remodeling the stromal microenvironment to facilitate immune infiltration. These coordinated mechanisms provide a scientific basis for combining SABR and LDRT to amplify systemic antitumor immunity.

## Ethical Approval

The multicenter study protocol was reviewed and approved by the Ethics Committee of The First Affiliated Hospital of University of Science and Technology of China (USTC; approval number: 2022ky-017) and the Ethics Committee of Hefei First People’s Hospital (approval number: 2022-007). Participating sites consisted of The First Affiliated Hospital of USTC, Hefei First People’s Hospital, Anhui Provincial Cancer Hospital, and Guoyang People’s Hospital. As Anhui Provincial Cancer Hospital and Guoyang People’s Hospital belong to the medical consortium led by The First Affiliated Hospital of the USTC, the ethical approval issued by the lead center (approval number: 2022ky-017) was mutually recognized by these 2 affiliated institutions. Written informed consent was obtained from all enrolled participants. This study was conducted following the Declaration of Helsinki and Good Clinical Practice. The animal study was conducted in accordance with the Institutional Animal Care Committee of The First Affiliated Hospital of University of Science and Technology of China (approval number: 2025-N (A)-071).

## Data Availability

The datasets generated during the study are publicly available in the Genome Sequence Archive database (https://ngdc.cncb.ac.cn/gsa) under accession number CRA035755. The publicly available GSE127465 dataset analyzed in this study is available from the Gene Expression Omnibus (GEO; https://www.ncbi.nlm.nih.gov/geo/). Bulk RNA-sequencing and clinical data from the TCGA-LUAD and TCGA-LUSC cohorts were obtained from TCGA (https://www.cancer.gov/tcga). Additional relevant data are available from the corresponding authors upon reasonable request.
