# Supplementary material for: Combined Immunotherapy and Hybrid Dose-Fraction Radiotherapy for Driver-Negative Metastatic NSCLC: A Phase II Trial with Parallel Preclinical Studies
Source: Cancer Commun (Lond). 2026 Nov 10;46:0065. doi: 10.34133/cancomm.0065 (PMC13748629; doi:10.34133/cancomm.0065)
Supplement: Supplementary file 1 [file cancomm.0065.f1.zip › CANCOMM-D-26-00363-Supplementary-Final.docx]

**Supplementary Materials for**

**Combined Immunotherapy and Hybrid Dose-Fraction Radiotherapy for Driver-Negative Metastatic NSCLC: A Phase II Trial with Parallel Preclinical Studies**

Ziye Li^1, 2†^, Xiaoyang Li^1, 3†^, Xiaoying Wei^1, 3^, Lejie Cao^2, 4^, Junqiang Zhang^2, 4^, Jianguo Wang^1, 2^, Yuan He^1, 3^, Anqi Hu^1^, Lei Liu^1^, Bing Yan^1^, Congyu Wang^5^, Hongxia Li^6^, Ping Zeng^6^, Jingjing Cheng^1, 2, 3^, Anlin Wang^1, 2^, Jun Ma^1, 3^, Huailing Xia^4^, Haibo Wu^2, 7^, Daqing Xia^4^, Fengshou Jiang^2, 8^, Dan Xie^9*^, and Dong Qian^1, 2, 3, 10*^

^1^ Department of Radiation Oncology, The First Affiliated Hospital of University of Science and Technology of China, Division of Life Sciences and Medicine, University of Science and Technology of China, Hefei, Anhui, P. R. China.

^2^ Core Facility Center, The First Affiliated Hospital of University of Science and Technology of China, Division of Life Sciences and Medicine, University of Science and Technology of China, Hefei, Anhui, P. R. China.

^3^ Anhui Provincial Cancer Hospital, Hefei, Anhui, P. R. China.

^4^ Department of Respiratory and Critical Care Medicine, The First Affiliated Hospital of University of Science and Technology of China, Division of Life Sciences and Medicine, University of Science and Technology of China, Hefei, Anhui, P. R. China.

^5^ The Center of Radiation Oncology, Guoyang County People’s Hospital, Bozhou, Anhui, P. R. China.

^6^ Department of Oncology, The Third Affiliated Hospital of Anhui Medical University, Hefei, Anhui, P. R. China.

^7^ Department of Pathology, The First Affiliated Hospital of University of Science and Technology of China, Division of Life Sciences and Medicine, University of Science and Technology of China, Hefei, Anhui, P. R. China.

^8^ Department of Medical Oncology, The First Affiliated Hospital of University of Science and Technology of China, Division of Life Sciences and Medicine, University of Science and Technology of China, Hefei, Anhui, P. R. China.

^9^ State Key Laboratory of Oncology in South China, Guangdong Provincial Clinical Research Center for Cancer, Sun Yat-sen University Cancer Center, Guangzhou, Guangdong, P. R. China.

^10^ Anhui Provincial Engineering Research Center for Radiation Oncology (AERRO), Hefei, Anhui, P. R. China.

^*^Corresponding Authors:

D.Q. (Dong Qian), E-mail: qiandong@ustc.edu.cn; D.X. (Dan Xie), E-mail: [xiedan@sysucc.org.cn](mailto:xiedan@sysucc.org.cn).

^†^These authors contributed equally to this work.

**Supplementary Figures**

**Supplementary Figure S1. Sensitivity and subgroup survival analyses. (A and B)** Kaplan–Meier curves of **(A)** PFS and **(B)** OS according to whether patients were downstaged to stage III after chemoimmunotherapy (Yes, *n* = 14; No, *n* = 69). **(C)** Kaplan–Meier curves of OS in PD-L1 TPS ≥50% (*n* = 14) vs. <50% (*n* = 61). **(D)** Kaplan–Meier curves of OS in PD-L1 subgroups: <1% (*n* = 24), 1%–49% (*n* = 37), ≥50% (*n* = 14). **(E)** Kaplan–Meier curves of OS in squamous (*n* = 39) vs. non-squamous (*n* = 44) carcinoma. **(F and G)** Kaplan–Meier curves of **(F)** PFS and **(G)** OS stratified by SABR coverage (≥50%, *n* = 37 *vs.* <50% of total lesions, *n* = 46). **(H and I)** Kaplan–Meier curves of **(H)** PFS and **(I)** OS stratified by metastatic burden (oligo-metastatic ≤3 lesions, *n* = 56 *vs.* poly-metastatic >3 lesions, *n* = 27). **(J and K)** Kaplan–Meier curves of **(J)** PFS and **(K)** OS stratified by liver metastasis status (with, *n* = 15 vs. without, *n* = 68). **(L and M)** Kaplan–Meier curves of **(L)** PFS and **(M)** OS for participants receiving different ICIs: pembrolizumab (*n* = 21), tislelizumab (*n* = 31), camrelizumab (*n* = 11), and sintilimab (*n* = 20). Statistical significance was assessed by log-rank test, with HR calculated via Cox proportional hazards model. **Abbreviations:** CI, confidence interval; HR, hazard ratio; ICI, immune checkpoint inhibitor; metas., metastases; OS, overall survival; PD-L1, programmed death-ligand 1; PFS, progression-free survival; SABR, stereotactic ablative body radiotherapy; TPS, tumor proportion score; *vs.*, versus.

**Supplementary Figure S2. Peripheral blood lymphocyte subsets in patients.**

**(A to E)** Paired scatter plots of peripheral blood lymphocyte subsets before and after Hybrid-RT (*n* = 40): **(A)** CD3⁺ T cells, defined as CD45⁺CD3⁺ cells; **(B)** Th cells, defined as CD3⁺CD4⁺ cells; **(C)** GZMB⁺ Th cells, defined as CD3⁺CD4⁺GZMB⁺ cells; **(D)** CD8⁺ T cells, defined as CD3⁺CD8⁺ cells; and **(E)** CD56ᵈⁱᵐ NK cells, defined as CD45⁺CD3⁻CD56ᵈⁱᵐ cells. Each dot represents an individual patient; lines connect paired pre- and post-RT measurements from the same patient. *P* values were determined by the paired *t* test. ns, not significant; **P* < 0.05; ****P* < 0.001. **Abbreviations:** CD3, cluster of differentiation 3; CD4, cluster of differentiation 4; CD45, cluster of differentiation 45; CD56, cluster of differentiation 56; CD8, cluster of differentiation 8; GZMB, granzyme B; Hybrid-RT, hybrid dose-fraction radiotherapy; NK, natural killer; RT, radiotherapy; Th, T helper.

**Supplementary Figure S3. Antitumor efficacy of IHBRT in NSCLC murine model. (A)** KEGG pathway enrichment heatmap of LDRT dose-response, (*n* = 3/group). **(B)** Average body weight of mice in indicated groups. **(C)** Masson staining of lung tissues (left) and quantification of collagen volume (right). **(D)** TGF-β IHC staining (left) and quantification of positive area (right). **(E)** GSEA enrichment plot of TGF-β signaling pathway (IHBRT vs. ISABR). **(F)** GSEA enrichment plot of ECM-receptor interaction pathway (IHBRT vs. ISABR). Data are presented as mean ± SD. ns, not significant. **Abbreviations:** anti-PD-1, anti-programmed cell death protein-1 antibody; ECM, extracellular matrix; ES, enrichment score; FDR, false discovery rate; GSEA, gene set enrichment analysis; ICI, immune checkpoint inhibitor; IHBRT, ICI plus Hybrid-RT; IHC, immunohistochemistry; ISABR, ICI plus SABR; KEGG, Kyoto Encyclopedia of Genes and Genomes; LDRT, low-dose radiotherapy; NES, normalized enrichment score; NSCLC, non-small cell lung cancer; SABR, stereotactic ablative body radiotherapy; SD, standard deviation; TGF-β, transforming growth factor beta.

**Supplementary Figure S4. T/NK subset enrichment revealed by single-cell sequencing.** **(A)** CNV analysis of scRNA-seq data from lung tissue in the Control, anti-PD-1, ISABR, and IHBRT groups (*n* = 3/group). **(B)** Bubble plot showing the average expression and expressed percentage of representative marker genes for different cell types in T/NK subtype. **(C and D)** Heatmap showing number **(C)** of ligand-receptor pairs and interaction strength **(D)** between T/NK subtype and tumor cells. **(E)** Bubble plot showing CCL and CXCL signaling pathway interactions between cyto-CD8⁺ T cells and other subtypes (IHBRT vs. ISABR). Bubble size indicates statistical significance (*P* < 0.01), and color intensity reflects the communication probability. **(F)** Violin plots showing *Cxcl10* expression across all subtypes in the IHBRT and ISABR groups. ns, not significant; ***P* < 0.01; ****P* < 0.001. **Abbreviations:** CCL, C-C motif chemokine ligand; *Cc*l, C-C motif chemokine ligand; CNV, copy number variation; CXCL, C-X-C motif chemokine ligand; cyto-CD8^+^, cytotoxic CD8^+^ T cell; *Cxcr3*, C-X-C motif chemokine receptor 3; ICI, immune checkpoint inhibitor; IHBRT, ICI plus Hybrid-RT; ILC2, group 2 innate lymphoid cell; ISABR, ICI plus SABR; NK, natural killer cell; NKT, natural killer T cell; ns, not significant; SABR, stereotactic ablative body radiotherapy; scRNA-seq, single-cell RNA sequencing; Treg, regulatory T cell.
